# Mitofusins are required for specialized mitochondrial morphology and function of rod photoreceptor cells

**DOI:** 10.3389/fcell.2026.1724328

**Published:** 2026-06-03

**Authors:** Michael Landowski, Ryo Hagimori, Purnima Gogoi, Pawan K. Shahi, Kazuya Oikawa, Vijesh J. Bhute, Gillian J. McLellan, Sakae Ikeda, Ken-ichi Yamada, Bikash R. Pattnaik, Tetsuya Takimoto, Akihiro Ikeda

**Affiliations:** 1 Department of Medical Genetics, University of Wisconsin-Madison, Madison, WI, United States; 2 McPherson Eye Research Institute, University of Wisconsin-Madison, Madison, WI, United States; 3 Department of Molecular Pathobiology, Faculty of Pharmaceutical Sciences, Kyushu University, Fukuoka, Japan; 4 Department of Pediatrics, University of Wisconsin-Madison, Madison, WI, United States; 5 Department of Ophthalmology and Visual Sciences, University of Wisconsin-Madison, Madison, WI, United States; 6 Department of Surgical Sciences, University of Wisconsin-Madison, Madison, WI, United States; 7 Department of Chemical Engineering, Imperial College London, South Kensington, London, United Kingdom; 8 Oncology Innovation Center, Fujita Health University, Toyoake, Aichi, Japan

**Keywords:** metabolic alteration, mitochondria, morphology, retina, rod photoreceptor cells

## Abstract

Mitochondria are dynamic organelles that undergo continuous morphological changes, yet exhibit unique, cell-type-specific structures. In rod photoreceptor cells of the retina, these include elongated mitochondria in the inner segments and a distinct, large, circular mitochondrion within each presynaptic terminal. The mechanisms underlying the establishment and maintenance of these specialized mitochondrial morphologies, as well as their relationship to photoreceptor function, remain incompletely understood. Here, we investigated the roles of mitochondrial fusion proteins mitofusin 1 (MFN1) and mitofusin 2 (MFN2) in rod photoreceptor cells. Rod-specific ablation of MFN1 and MFN2 resulted in near-complete and uniform mitochondrial fragmentation by 1 month of age, indicating that mitochondrial fusion is required for the development and maintenance of photoreceptor cell-specific mitochondrial architecture. At this stage, the layer structures of the retina examined by light microscopy appeared largely unaffected. Despite the absence of overt structural degeneration, electroretinography revealed early functional impairment, including reduced a-wave amplitudes and attenuation of the c-wave, indicating compromised rod photoreceptor activity and disrupted photoreceptor–RPE interactions. This was followed by progressive photoreceptor cell degeneration observed at 2 and 3 months of age. MFN1/2 ablation was also associated with changes in proteins involved in glycolysis, oxidative phosphorylation, and β-oxidation, along with activation of cellular stress pathways, including ER stress and the unfolded protein response. While total retinal ATP levels were only modestly reduced at early stages, these findings are consistent with alterations in metabolic homeostasis. Together, our findings demonstrate that MFN1 and MFN2 are required for specialized mitochondrial architecture in rod photoreceptor cells, and that their loss is associated with molecular remodeling and early functional deficits, preceding progressive degeneration.

## Introduction

Mitochondria, the energy-generating organelles, dynamically fuse and fission to take various forms in cells. Mitochondrial structure is generally dynamic in nature, and some cells display very unique mitochondrial structures. However, the exact relationship between their unique form and associated function are still not fully understood. Retinal rod photoreceptor cells, the energy-intensive neurons, provide an excellent model for investigating the significance of mitochondrial form and its function since they exhibit a uniquely uniform arrangement of elongated mitochondria in the inner segments and one large circular mitochondrion in each of the presynaptic terminals (Meschede et al., 2020; Ozaki et al., 2020; Ibrahim et al., 2025).

Energy homeostasis in rod photoreceptor cells is unique in that they use more than 80% of glucose for aerobic glycolysis converting it to lactate, rather than complete respiration including glycolysis, tricarboxylic acid (TCA) cycle, and oxidative phosphorylation (OXPHOS) (Ames 3rd et al., 1992; Pan et al., 2021). Nonetheless, they do rely on OXPHOS for energy production since less than 20% of glucose that enters OXPHOS has been proposed to account for 80% of the total ATP generation (Pan et al., 2021). Energy production in rod photoreceptor cells occurs mainly in the inner segments (Linton et al., 2010), where the glycolytic system is dominant (Pan et al., 2021; Chen Y. et al., 2023) and numerous characteristically elongated mitochondria are present (Meschede et al., 2020; Ozaki et al., 2020). Synapses also require large amounts of energy to circulate neurotransmitters, which is maintained by local OXPHOS and glycolysis (Li and Sheng, 2022). While mitochondria in rod photoreceptor cells show unique localization and morphology, it is still not completely clear how mitochondrial architecture contributes to the regulation of glycolysis and OXPHOS, and to the homeostasis of rod photoreceptor cells.

Regulation of mitochondrial structure has been linked to complex cellular processes such as metabolism, immune response, and cell death. Mitochondria maintain ATP-producing capacity and homeostasis through fission and fusion, and the balance of mitochondrial fission and fusion is tightly regulated in accordance with cellular metabolic states (Youle and Van Der Bliek, 2012). Impaired mitochondrial fission and fusion can disrupt metabolic homeostasis and ultimately compromise cell viability (Muñ oz-Pinedo et al., 2006; Chen W. et al., 2023). Mitochondrial fusion is stimulated by high-energy demand (Youle and Van Der Bliek, 2012). Mitofusin (MFN) 1 and MFN2 contribute to mitochondria fusion and regulate proper mitochondrial structure (Schrepfer and Scorrano, 2016; Tilokani et al., 2018). Studies have shown that MFN1 or MFN2 deficiency results in abnormal energy production and defective biosynthetic processes (Dai and Jiang, 2019). For example, it has become clear using gene targeted mice that mitochondrial fusion in pro-opiomelanocortin neurons regulates intracellular metabolism and maintains its function robustly (Schneeberger et al., 2013; Ramírez et al., 2017). However, the role of mitochondrial fusion in photoreceptor cell-specific structures and metabolic activities is still to be determined.

In this study, we ablated MFN1 and MFN2 specifically in rod photoreceptor cells to determine the role of mitochondrial fusion in maintaining their unique architecture and function *in vivo*. Loss of MFN1/2 resulted in marked mitochondrial fragmentation within the inner segments and synaptic terminals of rod photoreceptor cells by 1 month of age, indicating that mitochondrial fusion is required for maintaining rod photoreceptor cell-specific mitochondrial structures. Notably, the layer structures of the retina examined by light microscopy appeared unaffected at this age. However, functional assessment revealed early impairment of rod responses prior to overt degeneration. Following this period, progressive photoreceptor cell degeneration was observed at 2 and 3 months of age. We further found that impaired mitochondrial fusion was associated with changes in the levels of proteins involved in OXPHOS and mitochondrial β-oxidation, consistent with alterations in metabolic pathways. Additionally, our multi-omics analysis revealed activation of cellular stress pathways and cytoprotective responses. Together, these findings suggest that mitochondrial architecture maintained through mitochondrial fusion is closely associated with rod photoreceptor function and cellular integrity.

## Materials and methods

### Animals

*Rho-Cre* mice (B6.Cg-*Pde6b* ^*+*^ Tg [Rho-icre]1Ck/Boc [JAX stock #015850, RRID: IMSR_JAX:015850]) (Chen et al., 2007), *Mfn1* floxed mice (*Mfn1* ^*flx/flx*^; B6.129 [Cg]-*Mfn1* ^*tm2Dcc*^/J [JAX stock #026401, RRID: IMSR_JAX:026401]) (Chen et al., 2007), and *Mfn2* floxed mice (*Mfn2* ^*flx/flx*^; B6.129 [Cg]-*Mfn2* ^*tm3Dcc*^/J [JAX stock #026525, RRID: IMSR_JAX:026525]) (Chen et al., 2007) were purchased from The Jackson Laboratory. All the strains were congenic on the C57BL/6 J background and tested negative for *Pde6b* ^*rd1*^ and *Crb1* ^*rd8*^ mutations. They were bred together to generate Rho-icre*/Mfn1* ^*flx/flx*^ */Mfn2* ^*flx/flx*^ mice on the C57BL/6J background used in this study. WT mice on the C57BL/6 J background were used as controls for these experiments. All animals were housed in the same animal facility at the University of Wisconsin-Madison under standardized environmental conditions, including a 12 h light/12 h dark cycle (lights on at 6:00 a.m. and off at 6:00 p.m.). Both male and female mice at one, two, and 3 months of age were used. Unless otherwise specified, all tissue collection and related experimental procedures were performed during the light phase within a defined midday time window (approximately 11:00 a.m. to 1:00 p.m.) to minimize potential circadian variability. All experiments were conducted in accordance with the National Institute of Health Guide for the Care and Use of Laboratory Animals and were approved by the Animal Care and Use Committee at the University of Wisconsin-Madison. The results and methods in this study are reported in accordance with the ARRIVE guidelines.

### Electron microscopy

Eyes were fixed with 2% paraformaldehyde (PFA) and 2% glutaraldehyde and submitted to the Electron Microscope Core at the University of Wisconsin-Madison for transmission EM processing as previously described (Johnson et al., 2006; Lee et al., 2016; Lewis et al., 2018). Eye sections were mounted on a 400-mesh thin bar grid, and images were collected where the grid bars intersected the neural retinas using a Phillips CM120 STEM microscope (FEI Company, Hillsboro, OR, USA) at ×8,800 magnification. Mitochondria numbers were counted using NIH’s ImageJ software. Rod photoreceptor synaptic terminals were identified based on established ultrastructural features, including a single synaptic ribbon and characteristic vesicle organization.

### Immunohistochemistry

Eyes were punctured with a needle in the cornea and fixed with 4% paraformaldehyde (PFA) for 30 min at room temperature. Then the cornea and lens were removed, and neural retinas were separated from the eyecups. Neural retinas were blocked in 10% normal donkey serum for 30 min at room temperature. Next, they were incubated overnight with the 1:50 diluted primary antibody against TOMM20 (Santa Cruz Biotechnology, Hercules, TX, USA; catalog # sc-17764; RRID: AB_62838) at 4 °C with slow shaking. They were rinsed in PBS, and incubated with a 1:250 diluted Donkey Anti-Mouse IgG H&L (Alexa Fluor® 488) (Abcam, Cambridge, United Kingdom; catalog # ab150105; RRID: AB_2732856) for 45 min at room temperature. Before mounting, four small incisions were made to permit flattening of the retina. Retinal whole mounts were imaged to visualize mitochondria in the inner segment using SoRa/W1 Spinning Disk Microscope (Nikon, Tokyo, Japan) at a ×100 magnification.

### Histological analysis

Eyes were fixed with 2% PFA and 2% glutaraldehyde overnight. Eyes were then rinsed with PBS and embedded in paraffin. Samples were submitted to Translational Research Initiatives in Pathology (TRIP) core at the University of Wisconsin-Madison for processing and sectioning. Six μm sections were cut on a RM 2135 microtome (Leica Microsystems, Wetzlar, Germany). Paraffin sections were stained with hematoxylin and eosin (H&E) using standard protocols to visualize retinal layers and imaged using an Axio Imager 2 microscope (Carl Zeiss MicroImaging, NY, USA) at a ×40 magnification.

### Electroretinograms

Full-field electroretinography (ERG) was performed on 1- and 2-month-old *Rho-cre/Mfn1* ^*flx/flx*^ */Mfn2* ^*flx/flx*^ mice and age-matched WT controls as previously described (Jiao et al., 2022). Mice were dark-adapted overnight and anesthetized with ketamine (80 mg/kg) and xylazine (16 mg/kg). Pupils were dilated with 1% tropicamide (Bausch + Lomb® Rx Pharmaceuticals, Bridgewater, NJ). Recordings were obtained using the Espion E2 system (Diagnosys, Lowell,MA) with a corneal contact lens electrode and hypromellose (Gonak, Akorn Pharmaceuticals, Lake Forest, IL) to maintain hydration and electrical contact. Reference and ground electrodes were placed subcutaneously in the cheek and tail, respectively. Body temperature was maintained at 37 °C. Scotopic responses were recorded under dark-adapted conditions (0 cd s/m^2^), and photopic responses were obtained after 10 min of light adaptation (30 cd/m^2^ background). Flash intensities ranged from 0.03 to 30 cd s/m^2^, with ten averaged responses per intensity. Interstimulus intervals were 2 s (0.03–3 cd s/m^2^) and 5 s (10–30 cd s/m^2^). RPE function was assessed by measuring c-wave amplitudes at 2.5 or 25 cd s/m^2^ with a 4-s acquisition time. All ERG recordings were performed during the light phase within a consistent time window (12:00 PM–4:00 PM), including follow-up recordings on the same mice. Control and experimental groups were assessed within the same time frame to minimize potential circadian variability. Data were extracted using Espion V6 software and analyzed in Origin 2023b (OriginLab, Northampton, MA). Results are presented as mean ± SEM, and statistical comparisons were performed using two-tailed Student’s t-tests.

### Bulk RNA-sequencing

Neural retinas were collected and pooled from individual 1-month-old WT and *Rho-cre/Mfn1* ^*flx/flx*^ */Mfn2* ^*flx/flx*^ mice. Samples were flash frozen and then submitted to GENEWIZ from Azenta Life Sciences (South Plainfield, NJ, USA) for processing. Total RNA was extracted from neural retinas with an RNeasy Plus Universal Mini kit (Qiagen, Hilden, Germany) following the Manufacturer’s protocols. Total RNA samples were quantified using a Qubit 2.0 Fluorometer (Life Technologies, Carlsbad, CA, USA), and RNA integrity was examined using a TapeStation 4200 (Agilent Technologies, Palo Alto, CA, USA). RNA sequencing libraries were prepared using the NEBNext Ultra RNA Library Prep Kit for Illumina (NEB, Ipswich, MA, USA) following the manufacturer’s instructions. Briefly, messenger RNAs were first enriched with Oligo (dT) beads. Enriched mRNAs were fragmented for 15 min at 94 °C. First-strand and second-strand cDNAs were subsequently synthesized. cDNA fragments were end-repaired and adenylated at 3′ends, and universal adapters were ligated to cDNA fragments, followed by index addition and library enrichment by limited-cycle PCR. The sequencing libraries were validated on the Agilent TapeStation and quantified by using Qubit 2.0 Fluorometer as well as by quantitative PCR (KAPA Biosystems, Wilmington, MA, USA). The sequencing libraries were clustered on a flow cell. After clustering, the flowcell was loaded on the Illumina HiSeq instrument (4000 or equivalent) according to the manufacturer’s instructions. The samples were sequenced using a 2 × 150 bp Paired End (PE) configuration. Image analysis and base calling were conducted by the HiSeq Control Software (HCS). Raw sequence data (.bcl files) generated from Illumina HiSeq were converted into fastq files and de-multiplexed using Illumina’s bcl2fastq 2.17 software. The RNA-Seq raw sequence files from this study are available on the Gene Expression Omnibus (GEO), accession number GSE297370.

### RNA-sequencing analysis

Gene expression read counts were analysed using NetworkAnalyst 3.0 (Zhou et al., 2019). *M. musculus* organism was selected with a bulk sequencing analysis workflow. The quality control step involved filtering genes with very high variance across samples. Genes were ranked based on variance, and those genes that ranked in the bottom 15% of the percentile were filtered out. Low-abundance genes below a threshold were also filtered out. Data was normalized using log 2 counts per million normalization method. Differential gene expression analysis was performed using EdgeR (Robinson et al., 2009). Gene set enrichment analysis was performed using WebGestalt (Liao et al., 2019) and different functional databases including Gene Ontology, KEGG, and WikiPathways were used for analysis. To further restrict the number of gene sets due to overlap of the genes, affinity cluster algorithm (Chong et al., 2018) was applied. Signalling pathway analysis was conducted on differentially expressed genes using the SIGNOR 2.0 database (Licata et al., 2020).

### Metabolomics

Neural retina tissues were isolated from mice and stored at −80 °C. These samples were submitted to Metabolomics Core Resource Laboratory at New York University. Each tissue sample was then weighed and transferred into a bead blaster tube on dry ice. Prior to extraction, 80% methanol in water containing the internal standard (AA standard) was placed on dry ice for approximately 15 min.

For homogenization and extraction, 100 μL of glass beads was added to each bead blaster tube containing the tissue sample, followed by the addition of 80% methanol in water with the AA standard to achieve a final tissue concentration of 10 mg/mL. The samples were homogenized using a bead blaster for 10 cycles, with each cycle consisting of 30 s on and 30 s off. Following homogenization, the samples were centrifuged at 21,000 xg for 3 min. A total of 450 μL of the supernatant was then collected from each. These collected supernatants were dried down using a SpeedVac, after which the dried sample was reconstituted in 50 μL of mass spectrometry-grade (MA grade) water. The reconstituted sample was sonicated for 2 min and subsequently centrifuged at 21,000 xg for 3 min. Finally, 20 μL of the processed sample was transferred into a glass insert within a 2 mL glass vial for analysis. Samples were analyzed with the hybrid LCMS assay after scaling the metabolite extraction to a measured aliquot (10 mg/mL) for each sample and metabolites were quantified. Overall, coverage of the library was 147 metabolites being detected. The resulting data were analyzed by principal components analysis (PCA), visualizing clusters, volcano plots, and other statistical comparisons. Data files have been uploaded to MetaboLights database (ID: MTBLS12512), http://www.ebi.ac.uk/metabolights/.

### ATP measurements

ATP levels were quantified using the ATP Determination Kit following the manufacturer’s instructions (Invitrogen™, Thermo Fisher Scientific, Waltham, MA; catalog #A22066). Briefly, retinal tissues were rapidly dissected, immediately snap-frozen, and kept in liquid nitrogen to minimize ATP degradation. At the time of assay, tissues were thawed on ice and homogenized in ice-cold ATP extraction buffer (100 mM Tris-HCl, pH 7.8, containing 4 mM EDTA). Homogenates were centrifuged at 12,000 × g for 5 min at 4 °C, and supernatants were collected for analysis. ATP values were normalized to the WT group to allow relative comparison across samples. ATP standards were included as positive controls to confirm proper assay performance.

### Western blot analysis

Tissues were isolated from mice and stored at −80 °C. Neural retina lysates were homogenized using a Bel-Art Homogenizer system motor in RIPA buffer (Thermo Fisher Scientific, Waltham, MA; catalog # 89901) containing protease inhibitors (Roche, Base, Switzerland; catalog # 11836170001), respectively. Protein concentrations were quantified using a BCA Protein Assay Kit (Thermo Fisher Scientific, Waltham, MA; catalog # 23227). Equal protein amounts were aliquoted, reduced using XT Reducing Agent (Biorad, Hercules, CA; catalog # 1610792) for 7 minutes at 105 °C, and loaded onto 10% Bis-Tris Criterion XT gels (Biorad, Hercules, CA; catalog # 3450112) in MOPS buffer (Biorad, Hercules, CA; catalog #1610788) and transferred to nitrocellulose membranes (Biorad, Hercules, CA; catalog # 1620115) or Immun-Blot PVDF membranes for Protein Blotting (Biorad, Hercules, CA, catalog # 1620177). Membranes were blocked with milk or EveryBlot Blocking Buffer (Biorad, Hercules, CA; catalog # 12010020), and probed overnight with their respective primary antibody at 4 °C. The primary antibodies and their dilutions used in this study can be found in Table 1. Blots were washed with TBST buffer the next day and incubated with their corresponding secondary antibody. Secondary antibodies used in this study included donkey anti-rabbit IgG 680RD (LI-COR; catalog # 926-68073; RRID: AB_10954442), donkey anti-rabbit IgG 800CW (LI-COR; catalog # 926-32213; RRID: AB_621848), donkey anti-goat IgG 680RD (LI-COR; catalog # 926-68074; RRID: AB_2650427), goat anti-mouse IgG1 800CW (LI-COR; catalog # 926-32350; RRID: AB_2782997), goat anti-mouse IgG2a 800CW (LI-COR; catalog # 926-32351; RRID: AB_2782998), and goat anti-mouse IgM 800CW (LI-COR; catalog #925-32280, RRID: AB_2814919). Blots were washed again with TBST and imaged using the Odyssey Imaging System (LI-COR Biosciences, Lincoln, NE) and analyzed using NIH’s ImageJ (Bethesda, MD). Blots were stripped with Newblot Stripping Buffer (LI-COR Biosciences, Lincoln, NE) according to the manufacturer’s protocol and re-probed with another primary antibody in this study. All immunobands were normalized to the loading control on their respective immunoblot.

**TABLE 1:** Primary antibodies used in Western blot analyses.

Primary antibody (dilution)	Species	Catalog No.	Manufacturer	RRID
MFN1 (1:1000)	Rabbit	13798-1-AP	Proteintech	AB_2266318
MFN2 (1:1000)	Rabbit	9482	Cell signaling technology	AB_2716838
mTOR (1:750)	Rabbit	2983	Cell signaling technology	AB_2105622
p-mTOR (1:500)	Rabbit	2971S	Cell signaling technology	AB_330970
GAPDH (1:2000)	Goat	PLA0302	Sigma-aldrich	AB_3101987
PKM2 (1:1000)	Rabbit	15822-1-AP	Proteintech	AB_1851537
LDHA (1:1000)	Rabbit	19987-1-AP	Proteintech	AB_10646429
OXPHOS (1:1000)	Mouse	ab110413	Abcam	AB_2629281
SDHA (1:1000)	Rabbit	11998	Cell signaling technology	AB_2750900
CACT (1:1000)	Rabbit	19363-1-AP	Proteintech	AB_10642001
CPT2 (1:1000)	Rabbit	26555-1-AP	Proteintech	AB_2880551
FASN (1:1000)	Rabbit	ab22759	Abcam	AB_732316
PMP70 (1:1000)	Rabbit	ab3421	Abcam	AB_2219901
TUB (1:2000)	Mouse	3873	Cell signaling technology	AB_1904178

### RNAscope in situ hybridization

The RNAscope *in situ* hybridization (ISH) was performed as previously described by Oikawa et al. 2023 (Oikawa et al., 2023). ISH was carried out using RNAscope Fluorescent Multiplex Kit V2 (catalog # 323100; Advanced Cell Diagnostics [ACD], Newark, CA, USA) according to the manufacturer’s instructions with minor modifications. Briefly, 8 µm-thick tissue cryosections were air-dried for 30 min, washed in 0.01M PBS for 5 min, and treated with hydrogen peroxide for 10 min, all at room temperature, then incubated with protease IV for 30 min at 40 °C using the HybEZ II Hybridization System (ACD). Tissue sections were incubated with specific probes targeting mRNA: Mm-Mfn1 (catalog # 578731), Mm-Mfn2 (catalog # 578761), Mm-Ppib (positive control, catalog # 313911), and DapB (negative control [a bacterial gene], catalog # 310043). Probes were fluorescently labeled with TSA plus Cy3 (Akoya Biosciences, 1:1500) and counterstained with DAPI.

### Statistical analysis

All statistical tests were performed using Prism Software (GraphPad, San Diego, CA). Significance of the difference between groups was calculated by unpaired Student’s two-tailed t-test, for experiments comparing two groups, and one-way or two-way analyses of variance (ANOVA) with the Bonferroni-Dunn multiple comparison posttest for experiments comparing three or more groups using *p < 0.05, **p < 0.01, ***p < 0.001. ****p < 0.0001. All data are presented as the mean ± the standard deviation (s.d.) of three or more independent experiments, with three or more replicates per condition per experiment. P < 0.05 was considered to be statistically significant.

## Results

### MFN1 and MFN2 are required for development of rod-specific mitochondrial morphology and integrity of rod photoreceptor cells

**FIGURE 1:**
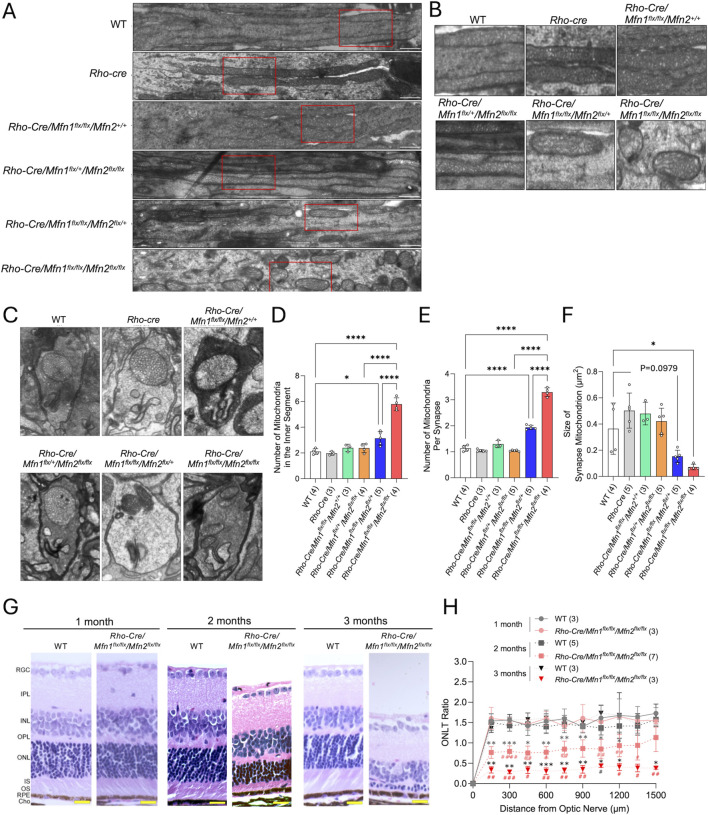
Abnormal mitochondrial morphologies in rod photoreceptor inner segments and synapses due to ablation of *Mfn1* and *Mfn2* at 1 month of age. **(A)** Representative electron micrographs of rod photoreceptor inner segments. Magnification = ×8,800. Scale bar = 1 micron. **(B)** Higher-magnification images of the regions outlined by red rectangles in panel **(A)**. **(C)** Representative electron micrographs of rod photoreceptor synapses. Magnification = ×8,800. **(D)** Quantification of the number of mitochondria in rod photoreceptor inner segments. **(E)** Quantification of the number of mitochondria in the rod photoreceptor synapse. **(F)** Quantification of the size of mitochondria in the rod photoreceptor synapse. Dots represent individual data points. Number in the parenthesis denotes the number of mice used in the experiment. Data is presented as mean±SD. *P < 0.05, ****P < 0.0001 by one-way ANOVA with *post hoc* Tukey’s test. **(G,H)** Photoreceptor cell degeneration in mice with rod-specific ablation of *Mfn1* and *Mfn2.* Representative images of H&E-stained retinal sections of 1-, 2- and 3-month-old mice. Magnification = ×40. Scale bar = 20 microns **(G)**. Outer nuclear layer thickness (ONLT) ratios **(H)**. Note that *Rho-Cre/Mfn1* ^*flx/flx*^ */Mfn2* ^*flx/flx*^ mice exhibit significant reduction of ONLT at 2 and 3 months of age. Number in the parenthesis denotes the number of mice used in the study. Data is presented as mean±SD. *P < 0.05, **P < 0.01, ***P < 0.001, ****P < 0.0001 by two-way ANOVA with *post hoc* Tukey’s test.

We ablated genes encoding mitochondrial fusion factors, MFN1 and MFN2, in murine rod photoreceptor cells by crossing mice with floxed alleles of *Mfn1* (*Mfn1* ^*flx*^, (Chen et al., 2007)) and *Mfn2* (*Mfn2* ^*flx*^, (Chen et al., 2007)) and *Rho-iCre* (*Rho-Cre*) transgenic mice, in which Cre-recombinase is expressed specifically in rod photoreceptor cells (Chen et al., 2007) (Supplementary Figure S1A). We confirmed a ∼10–15% reduction of MFN1 and MFN2 in the neural retina of these mice (*Rho-Cre/Mfn1* ^*flx/flx*^ */Mfn2* ^*flx/flx*^; Supplementary Figure S1B). This modest reduction reflects selective depletion in rod photoreceptor cells, while MFN1 and MFN2 remain expressed in other retinal cell types not targeted by Cre recombination. Consistent with this, RNAscope *in situ* hybridization (ISH) analysis demonstrated marked reduction of *Mfn1* and *Mfn2* transcripts specifically within the photoreceptor layer (Supplementary Figure S1C). To investigate the role of mitochondrial fusion in shaping the unique mitochondrial structures and function in rod photoreceptor cells, we examined mitochondrial morphologies and retinal health in mice with rod-specific deletion of MFN1 and MFN2 (*Rho-Cre/Mfn1* ^*flx/flx*^ */Mfn2* ^*flx/flx*^). Electron microscopy (EM) revealed significantly increased mitochondrial fragmentation in the inner segments of rod photoreceptor cells in *Rho-Cre/Mfn1* ^*flx/flx*^ */Mfn2* ^*flx/flx*^ mice at 1 month of age (Figures 1A,B,D; Supplementary Figure S3A). Consistent changes were observed by confocal imaging (Supplementary Figure S3B). EM analysis of rod photoreceptor synaptic terminals—identified based on characteristic features including a single synaptic ribbon and vesicle organization in rod spherules—revealed significantly increased mitochondrial fragmentation in *Rho-Cre/Mfn1* ^*flx/flx*^ */Mfn2* ^*flx/flx*^ mice at 1 month of age (Figures 1C,E; Supplementary Figure S2), accompanied by a significant decrease in mitochondrial size within the synapse (Figure 1F). While *Rho-Cre/Mfn1* ^*flx/flx*^ */Mfn2* ^*+/+*^ mice and *Rho-Cre/Mfn1* ^*flx/+*^ */Mfn2* ^*flx/flx*^ mice exhibit intact mitochondrial architecture, *Rho-Cre/Mfn1* ^*flx/flx*^ */Mfn2* ^*flx/+*^ mice showed moderately fragmented mitochondria in the synapse and inner segments (Figures 1A–F; Supplementary Figures S2, S3A). No changes in mitochondrial aspect ratio were observed in synapses of any of the genotypes (Supplementary Figure S1D). These findings demonstrate that MFN1 and MFN2-mediated mitochondrial fusion contributes to shaping the distinct mitochondrial architectures within rod photoreceptor cells.

Histological analysis at the light microscopy level showed no gross abnormalities in rod photoreceptor cells at 1 month of age across genotypes (Figures 1G,H). At 2 months of age, moderate photoreceptor degeneration was observed in *Rho-Cre/Mfn1* ^*flx/flx*^ */Mfn2* ^*flx/flx*^ mice, with approximately 50% reduction in outer nuclear layer (ONL) thickness (Figures 1G,H). By 3 months of age, more pronounced photoreceptor degeneration was evident in these mice (Figures 1G,H). These findings are consistent with an association between mitochondrial structural integrity and maintenance of rod photoreceptor integrity.

### Loss of mitochondrial fusion leads to early functional impairment in rod photoreceptors

**FIGURE 2:**
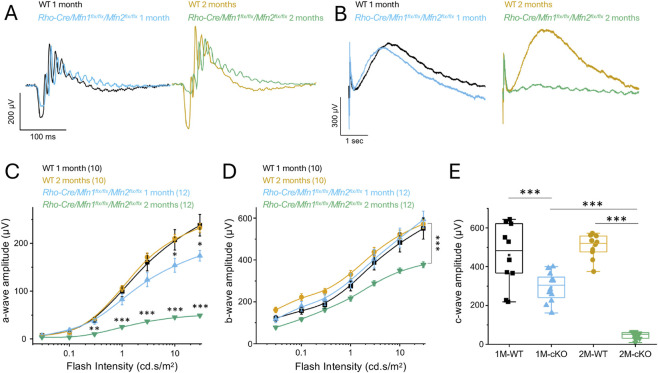
ERG analysis of *Rho-Cre/Mfn1* ^*flx/flx*^ */Mfn2* ^*flx/flx*^ mice and age-matched WT controls. **(A,B)** Representative scotopic (rod-driven) ERG traces showing a- and b-waves **(A)** and c-waves **(B)** from 1-month-old WT (black) and *Rho-Cre/Mfn1* ^*flx/flx*^ */Mfn2* ^*flx/flx*^ (blue) mice, and 2-month-old WT (yellow) and *Rho-Cre/Mfn1* ^*flx/flx*^ */Mfn2* ^*flx/flx*^ (green) mice. **(C–E)** Quantification of scotopic ERG responses. Compared to WT controls, *Rho-Cre/Mfn1* ^*flx/flx*^ */Mfn2* ^*flx/flx*^ mice exhibit a reduction in a-wave amplitude as early as 1 month of age **(C)** followed by a significant reduction in both a-wave and b-wave amplitude by 2 months **(D)**, and an age-dependent decline in c-wave amplitude **(E)**, consistent with progressive impairment of rod photoreceptor function and photoreceptor–RPE coupling. Number in the parenthesis denotes the number of mice used in the study. Data are presented as mean ± SEM, and statistical significance (*P < 0.05, **P < 0.01, ***P < 0.001) was determined using two-tailed Student’s *t*-tests.

To determine whether the mitochondrial architectural abnormalities observed in *Rho-Cre/Mfn1* ^*flx/flx*^ */Mfn2* ^*flx/flx*^ photoreceptors were accompanied by functional deficits, we performed full-field electroretinography (ERG) in *Rho-Cre/Mfn1* ^*flx/flx*^ */Mfn2* ^*flx/flx*^ mice and age-matched WT controls at one and 2 months of age (Figure 2) prior to the more severe degeneration observed at 3 months (Figures 1G,H). Under scotopic conditions, *Rho-Cre/Mfn1* ^*flx/flx*^ */Mfn2* ^*flx/flx*^ mice exhibited a significant reduction in a-wave amplitude at 1 month of age compared to WT controls (Figures 2A,C), despite preserved morphology of the photoreceptor cell layer at this stage as assessed by light microscopy of H&E–stained sections (Figures 1G,H). By 2 months, a-wave amplitudes were further significantly reduced relative to both age-matched WT controls and one-month-old *Rho-Cre/Mfn1* ^*flx/flx*^ */Mfn2* ^*flx/flx*^ mice (Figures 2A,C), consistent with progressive impairment of rod photoreceptor function. Scotopic b-wave amplitudes were also significantly decreased at 2 months in *Rho-Cre/Mfn1* ^*flx/flx*^ */Mfn2* ^*flx/flx*^ retinas (Figures 2A,D). Notably, the scotopic c-wave amplitude was already significantly reduced at 1 month of age (Figures 2B,E). By 2 months, the c-wave response was further reduced and approached near-flat levels compared to both 1-month-old *Rho-Cre/Mfn1* ^*flx/flx*^ */Mfn2* ^*flx/flx*^ mice and WT controls (Figures 2B,E). H&E staining at 2 months revealed moderate photoreceptor degeneration, with approximately half of the ONL remaining (Figures 1G,H), indicating that the marked reduction in c-wave amplitude is not fully accounted for by photoreceptor loss alone. Together, these data demonstrate that mitochondrial architectural disruption due to loss of mitofusins is associated with early functional impairment of rod photoreceptors prior to overt structural degeneration, accompanied by evidence of disrupted photoreceptor–RPE coupling and a subsequent progressive decline in rod-driven responses.

### Molecular pathways altered by ablation of *Mfn1* and *Mfn2*

To elucidate the molecular pathways associated with rod photoreceptor cell degeneration in *Rho-cre/Mfn1* ^*flx/flx*^ */Mfn2* ^*flx/flx*^ mice, we performed RNA sequencing (RNA-seq) on neural retinas collected from 1-month-old WT and *Rho-cre/Mfn1* ^*flx/flx*^ */Mfn2* ^*flx/flx*^ mice prior to overt photoreceptor cell degeneration. Differential expression analysis identified 974 dysregulated genes (399 upregulated and 575 downregulated) in *Rho-cre/Mfn1* ^*flx/flx*^ */Mfn2* ^*flx/flx*^ mice compared to WT controls, revealing substantial transcriptional changes.

In the neural retina of *Rho-cre/Mfn1* ^*flx/flx*^ */Mfn2* ^*flx/flx*^ mice, cell-specific markers (total 75) are not significantly changed or showed log2 fold changes (LogFC) < 1 overall except for 9 markers of macrophage, Müller cell, and oligodendrocyte (Supplementary Figure S4), consistent with minimal changes in cellular composition. This finding was consistent with the results of histological analysis by light microscopy (Figures 1G,H).

**FIGURE 3:**
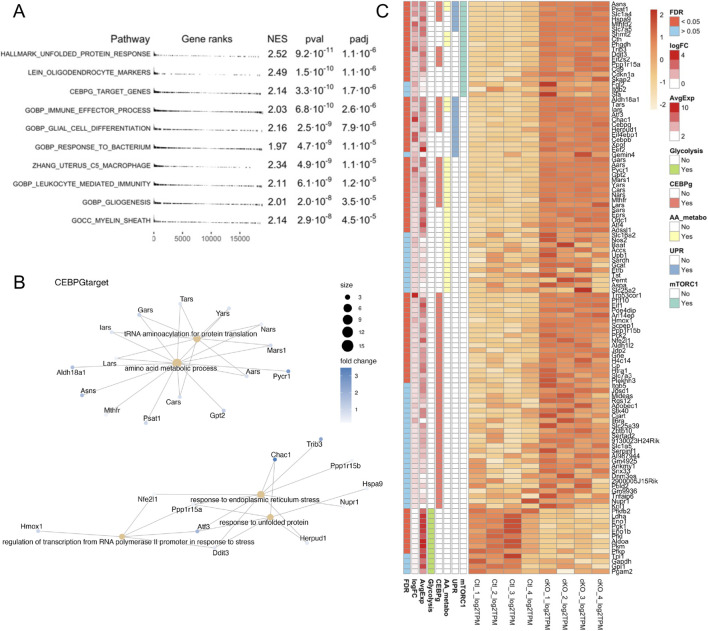
Molecular pathways altered in the neural retina by rod-specific ablation of *Mfn1* and *Mfn2*. **(A)** Top10 enriched pathways were identified by Gene Set Enrichment Analysis (GSEA) and ranked by P value (pval) and adjusted P value (padj). NES stands for normalized enrichment score. **(B)** Overrepresentation analysis of the CEBPG_TARGET_GENES gene sets with the highest expression variation ratio. **(C)** Heatmap showing genes involved in mTORC1 signaling, amino acid (AA) metabolism, unfolded protein response (UPR), and C/EBPγ that are significantly enriched, and glycolysis-related genes that are significantly downregulated in the neural retina by rod-specific ablation of *Mfn1* and *Mfn2*. False discovery rate (FDR), logFC, and average expression (AvgExpr) are shown in the left column for each gene.

To investigate the functional relevance of dysregulated genes, we conducted gene set enrichment analysis (GSEA) to identify the top 10 enriched gene sets, using LogFC values between WT and *Rho-cre/Mfn1* ^*flx/flx*^ */Mfn2* ^*flx/flx*^ (conditional KO [cKO]) retinas with the fast gene set enrichment analysis algorithm (Figure 3A). A positive normalized enrichment score (NES) indicated that genes upregulated in cKO retinas were predominantly enriched in the top-ranking gene sets. Pathway analysis revealed that genes involved in ‘unfold protein response (UPR)’ and ‘CCAAT/enhancer-binding protein gamma (C/EBPγ)’ (Figure 3A), both of which are known to respond to ER stress (Hetz, 2012; Huggins et al., 2016; Xu and Wang, 2024) were enriched. Furthermore, overrepresentation analysis of the genes in CEBPG_TARGET_GENES with the highest expression variation ratios confirmed the enrichment of genes related to amino acids (AA) metabolism and translation process in *Rho-cre/Mfn1* ^*flx/flx*^ */Mfn2* ^*flx/flx*^ mice (Figure 3B). Our GSEA results also revealed that the gene set involved in mammalian target of rapamycin complex 1 (mTORC1) signaling was enriched to the higher level of the gene set expression variability ratio in *Rho-cre/Mfn1* ^*flx/flx*^ */Mfn2* ^*flx/flx*^ mice (Figure 3C). Notably, in addition to these findings, several key glycolytic genes were suppressed (Figure 3C).

### Metabolomic changes resulting from ablation of *Mfn1* and *Mfn2*

**FIGURE 4:**
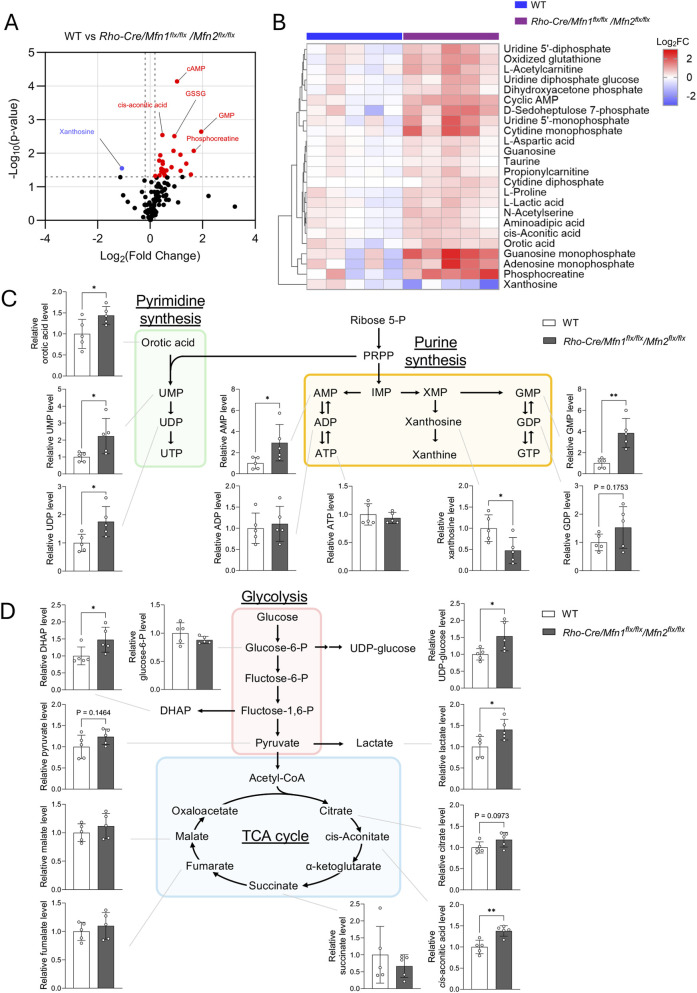
Metabolic changes in the neural retina resulting from rod-specific ablation of *Mfn1* and *Mfn2*. **(A)** Volcano plot showing differentially changed metabolites in the neural retina of *Rho-Cre/Mfn1* ^*flx/flx*^ */Mfn2* ^*flx/flx*^ mice versus WT mice. **(B)** Heatmap showing significantly changed metabolites in the neural retina of *Rho-Cre/Mfn1* ^*flx/flx*^ */Mfn2* ^*flx/flx*^ mice versus WT mice (P < 0.05). **(C)** Schematic diagram of pyrimidine and purine synthesis, and relative metabolite levels associated with nucleotide synthesis in *Rho-Cre/Mfn1* ^*flx/flx*^ */Mfn2* ^*flx/flx*^ neural retina compared to WT neural retina. **(D)** Schematic diagram of glycolysis and TCA cycle, and relative metabolite levels associated with these pathways in *Rho-Cre/Mfn1* ^*flx/flx*^ */Mfn2* ^*flx/flx*^ neural retina compared to WT neural retina. Data are presented as mean ± SD. Asterisks (*) indicate P < 0.05 significance by t-test. Five mice were used for each group in the study. Dots represent individual data points.

To investigate the difference of metabolites influenced by mitochondrial fragmentation in *Rho-cre/Mfn1* ^*flx/flx*^ */Mfn2* ^*flx/flx*^ mice, we conducted targeted metabolomics analysis on neural retinas collected from 1-month-old mice. Three-dimensional principal component analysis (3D-PCA) of the metabolomics profiles revealed clear separation between *Rho-cre/Mfn1* ^*flx/flx*^ */Mfn2* ^*flx/flx*^ groups and WT groups (Supplementary Figure S5A). In total, 147 metabolites were identified, among which 23 metabolites were significantly upregulated and one was downregulated (p < 0.05) (Figures 4A,B). Our metabolomics analysis revealed alterations in metabolites associated with purine, pyrimidine, and lactate synthesis pathways (Figures 4B–D). ATP levels showed a downward trend in *Rho-cre/Mfn1* ^*flx/flx*^ */Mfn2* ^*flx/flx*^ mice (Figure 4C; Supplementary Figure S5B). Moreover, several amino acids such as proline and aspartate were significantly upregulated (Figure 4B; Supplementary Figure S5C).

### Changes in protein levels in pathways associated with mitochondrial fusion deficiency

**FIGURE 5:**
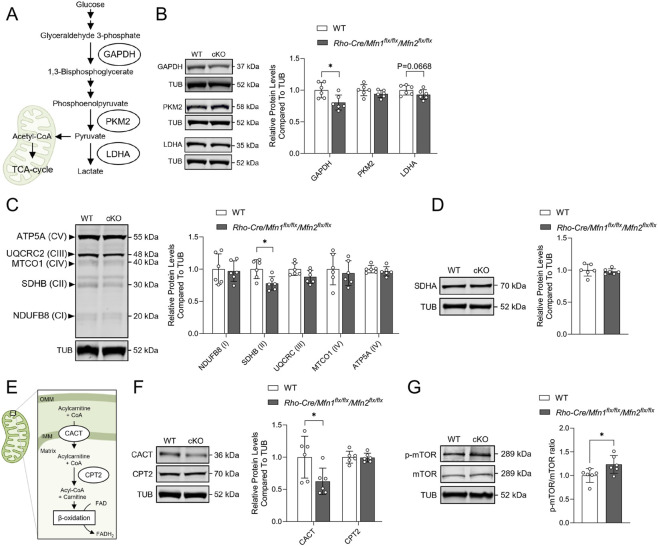
Identified changes in protein levels associated with pathways presumed to respond to mitochondrial fusion defects. **(A)** Schematic diagram of the glycolysis pathway of lactate synthesis from glucose through pyruvate in the cytosol of cells. **(B)** Western blot analysis of glyceraldehyde-3-phosphate dehydrogenase (GAPDH), pyruvate kinase M2 (PKM2), and lactate dehydrogenase (LDH), which are related to glycolysis pathway in the neural retina of *Rho-Cre/Mfn1* ^*flx/flx*^ */Mfn2* ^*flx/flx*^ mice versus WT mice. **(C)** Western blot analysis of each subunit comprising the complexes (Complex I (CI): NADH dehydrogenase [ubiquinone] 1 beta subcomplex subunit 8 (NDUFB8), Complex II (CII): succinate dehydrogenase B (SDHB), Complex III (CIII): ubiquinol-cytochrome c reductase core protein 2 (UQCRC2), Complex IV (CIV): mitochondrially encoded cytochrome c oxidase I (MTCO1), Complex V (CV): ATP synthase F1 subunit alpha (ATP5A)) responsible for oxidative phosphorylation (OXPHOS). **(D)** Western blot analysis of another CII subunit, succinate dehydrogenase A (SDHA). **(E)** Schematic diagram of the substrate uptake and pathway toward mitochondrial β-oxidation. **(F)** Western blot analysis of carnitine-acylcarnitine translocase (CACT) and carnitine palmitoyl transferase II (CPT2), involved in mitochondrial β-oxidation. **(G)** Western blot analysis of mammalian target of rapamycin (mTOR) and phosphorylated-mTOR-S2448 (p-mTOR). Protein levels of p-mTOR were normalized by that of mTOR. Alpha-tubulin (TUB) served as the loading control for this Western blot experiments except for the result of p-mTOR. Data are presented as mean ± SD. Asterisks (*) indicates P < 0.05 significance following a significant difference detected by t-test. Six one-month-old mice were used in both groups in study. Dots represent individual data points. The protein size next to the immunoblot images denotes the size of the immunobands measured for this analysis.

Our RNA-seq results indicated significant downregulation of genes involved in glycolysis (Figure 3C). Rod photoreceptor cells require substantial energy for their function (Narayan et al., 2017; Fu et al., 2021) and mainly utilize glucose for aerobic glycolysis converting glucose to pyruvate, which is then converted to lactate by lactate dehydrogenase A (LDHA) (Pan et al., 2021) (Figure 5A). Western blot analysis of proteins involved in glycolysis revealed a significant decrease in glyceraldehyde-3-phosphate dehydrogenase (GAPDH) expression, while pyruvate kinase M2 (PKM2) and LDHA levels remained unchanged (Figure 5B). Subsequent to glycolysis, pyruvate is also converted to acetyl-CoA to replenish the TCA cycle followed by OXPHOS for energy production in photoreceptor cells (Fu et al., 2021), and therefore, we examined the expression of OXPHOS complex subunits. Notably, succinate dehydrogenase complex iron sulfur subunit B (SDHB), a component of Complex II that oxidizes FADH_2_ to FAD, was significantly decreased in *Rho-cre/Mfn1* ^*flx/flx*^ */Mfn2* ^*flx/flx*^ mice (Figure 5C). SDHA, another subunit of Complex II responsible for supplying SDHB with FADH_2_ synthesized via the TCA cycle, remained unchanged (Figure 5D). In addition to the TCA cycle, mitochondrial β-oxidation serves as another source of FADH_2_ (Kemp et al., 2024). The mitochondrial β-oxidation pathway begins with the uptake of acyl-CoA into mitochondria via carnitine-acylcarnitine translocase (CACT), followed by conversion to acyl-CoA by carnitine palmitoyl transferase 2 (CPT2) (Kemp et al., 2024) (Figure 5E). Western blot analysis revealed a significant reduction in CACT expression, while CPT2 levels remained unchanged (Figure 5F). In contrast, the expression of 70-kDa peroxisomal membrane protein (PMP70), a transporter involved in peroxisomal β-oxidation, and fatty acid synthase (FASN), an enzyme responsible for synthesizing fatty acids that serve as substrates for peroxisomal β-oxidation remained unchanged in *Rho-cre/Mfn1* ^*flx/flx*^ */Mfn2* ^*flx/flx*^ mice (Supplementary Figure S6).

mTORC1 serves as a central regulator of cellular homeostasis by integrating diverse cellular signals (Saxton and Sabatini, 2017; Panwar et al., 2023). It is inhibited under energetic stress but becomes activated in response to amino acid availability and oxidative stress (Saxton and Sabatini, 2017; Panwar et al., 2023). Our RNA-seq analysis revealed that many genes related to mTORC1 pathway are upregulated, along with amino acid metabolism-related genes in *Rho-cre/Mfn1* ^*flx/flx*^ */Mfn2* ^*flx/flx*^ mice (Figure 3C). Western blot analysis showed elevated levels of phosphorylated-mTOR (p-mTOR) levels (Figure 5G).

## Discussion

In this study, we demonstrate that MFN1 and MFN2 are required for establishing rod photoreceptor cell-specific mitochondrial architecture. Loss of mitochondrial fusion is associated with disruption of this organization, accompanied by molecular remodeling, early functional impairment as measured by ERG, and progressive photoreceptor degeneration. Our findings define three key aspects of the phenotypes: structural alterations in mitochondrial morphology, changes in metabolic and stress-related pathways, and functional deficits assessed by ERG. These results provide a framework for understanding the effects of MFN1/2 ablation across mitochondrial architecture, cellular state, and photoreceptor function.

### Mitofusins regulate mitochondrial structures in rod photoreceptor cells

Rod photoreceptor cells exhibit highly specialized mitochondrial architecture, characterized by a large, singular mitochondrion at the synaptic terminal and elongated mitochondria in the inner segment. Our findings suggest that mitochondrial fusion is essential for establishing these unique structures (Figures 1A–C). Smaller mitochondria were observed in inner segments and synaptic terminals of *Rho-cre/Mfn1* ^*flx/flx*^ */Mfn2* ^*flx/flx*^ mice, suggesting that smaller mitochondria reach the synaptic terminal, where mitofusins may normally mediate their subsequent fusion into the distinctive morphologies. Interestingly, MFN2 has been implicated in mitochondrial trafficking into synaptic terminals in other neuronal cells (Misko et al., 2010; Vevea and Chapman, 2023). However, our results indicate that mitofusins may not play a direct role in mitochondrial trafficking in rod photoreceptor cells, as smaller mitochondria were observed at their destinations even in the absence of MFN2 or MFN1. This suggests a mitofusin-independent mechanism for mitochondrial trafficking in rod photoreceptor cells, whereas mitofusins primarily regulate mitochondrial morphology following arrival at their destinations.

### Coordination between MFN1 and MFN2

MFN1 and MFN2 are present on the outer mitochondrial membrane (OMM) and act cooperatively to regulate mitochondrial fusion (Santel and Fuller, 2001; Filadi et al., 2018; Tábara et al., 2025). Our EM analysis revealed that depletion of both MFN1 and MFN2 caused marked mitochondrial fragmentation (Figures 1A–C), suggesting that the specialized mitochondrial architecture of rod photoreceptor cells depends on mitochondrial fusion mediated by both proteins. *Rho-cre/Mfn1* ^*flx/+*^ */Mfn2* ^*flx/flx*^ mice and *Rho-cre/Mfn1* ^*flx/flx*^ */Mfn2* ^*+/+*^ mice exhibited proper development of mitochondrial morphologies (Figures 1A–C), indicating that MFN1 and MFN2 compensate for each other in terms of the formation of rod photoreceptor cell-specific mitochondrial morphologies. Consistent with our findings, a previous report showed that photoreceptor cell degeneration in MFN2-mutant mice (*MFN2* ^*R94Q*^) is rescued by augmentation of MFN1 (*MFN2* ^*R94Q*^:*MFN1*) (Shahin et al., 2023). Mammalian MFN1 and MFN2 are very similar proteins with high homology (∼80%) (Filadi et al., 2018; Tábara et al., 2025). Mitochondrial membrane fusion requires interaction of mitofusins with the C-terminal heptad repeat domain (HR2), and dimerization of the GTPase domain (Filadi et al., 2018). Given the high degree of homology between MFN1 and MFN2 in both their GTPase and HR2 domains, it is plausible that they may functionally compensate for each other. However, *Rho-cre/Mfn1* ^*flx/flx*^ */Mfn2* ^*flx/+*^ mice showed moderate mitochondrial fragmentation, while *Rho-cre/Mfn1* ^*flx/+*^ */Mfn2* ^*flx/flx*^ mice displayed intact mitochondria (Figures 1A–F). This observation suggests that the functions of MFN1 and MFN2 are not completely redundant in mitochondrial fusion. Several potential explanations may account for this observation. MFN1 has been shown to regulate inner mitochondrial membrane (IMM) fusion by controlling the expression levels of other mitochondrial dynamics proteins such as OPA1 and Fis1 (Shahin et al., 2023). Therefore, in addition to affecting mitochondrial fusion through functions that are shared by MFN2 (and thus, can be compensated for by MFN2), MFN1 may also regulate mitochondrial fusion through distinct, MFN1-specific functions/mechanisms. Furthermore, it has been reported that MFN1 possesses eightfold higher GTPase activity compared to MFN2, suggesting a potential difference in their abilities to drive mitochondrial fusion (Ishihara et al., 2004). In addition, the protein levels of MFN1 and MFN2 may influence mitochondrial fusion, which may be different in rod photoreceptor cells. Finally, both MFN1 and MFN2 need to be recruited to the mitochondria to exert their functions in mitochondrial fusion. This recruitment to the OMM is mediated through interactions between their N-terminal mitochondrial targeting sequences and mitochondrial translocation complexes (Bauer et al., 2000; Santel and Fuller, 2001). It is possible that differences exist between MFN1 and MFN2 in the process of mitochondrial recruitment.

### Rod photoreceptor functional impairment precedes structural degeneration

To determine how mitochondrial structural disruption relates to photoreceptor function, we analyzed ERG responses and found early functional impairment (Figure 2) prior to overt structural degeneration (Figures 1G,H). Specifically, reduced scotopic a-wave amplitudes (Figures 2A,C) indicate compromised rod photoreceptor activity. In addition, early and pronounced attenuation of the c-wave (Figures 2B,E) suggests disruption of photoreceptor–RPE interactions, which may reflect impaired communication between photoreceptors and the RPE. Notably, the magnitude of c-wave reduction at early time points exceeds what would be expected based on the degree of photoreceptor loss alone, indicating that functional impairment precedes overt structural degeneration. At early time points, relative preservation of scotopic b-wave responses (Figures 2A,D), which primarily reflect downstream bipolar cell activity, suggests partial maintenance of inner retinal signaling despite primary photoreceptor dysfunction. As degeneration progresses, reductions in b-wave amplitudes become more apparent, consistent with increasing disruption of rod-driven retinal circuitry. Together, these findings support a model in which mitochondrial structural disruption is associated with early functional deficits that precede overt photoreceptor cell loss.

### Altered metabolic state associated with mitochondrial fusion deficiency

Mitochondrial fusion and fission contribute to the maintenance of mitochondrial function by facilitating the exchange and distribution of mitochondrial components (Adebayo et al., 2021; Youle and Van Der Bliek, 2012). In *Rho-cre/Mfn1* ^*flx/flx*^ */Mfn2* ^*flx/flx*^ mice, we observed reduced levels of SDHB, a subunit of Complex II (CII) involved in oxidative phosphorylation (OXPHOS) (Figure 5C), as well as decreased CACT (Figure 5F), a transporter required for the import of acylcarnitines into mitochondria during β-oxidation (Kemp et al., 2024). These changes were accompanied by metabolomic alterations, including increased levels of acylcarnitines such as L-acetyl carnitine and propionyl carnitine (Figure 4B), consistent with altered regulation of mitochondrial lipid metabolism. Rod photoreceptors are highly metabolically active neurons that rely on coordinated glycolysis and mitochondrial metabolism to sustain their function (Narayan et al., 2017; Fu et al., 2021; Pan et al., 2021). While we observed changes in metabolic enzyme levels, including reduced GAPDH (Figure 5B), these findings do not directly demonstrate altered metabolic flux. Rather, they suggest remodeling of metabolic protein composition in response to mitochondrial structural disruption. Additional metabolomic changes, such as increased levels of L-proline and phosphocreatine (Figure 4B), are consistent with adaptive responses related to energy buffering and redox balance (Guimarães-Ferreira, 2014; Dash et al., 2021; Choudhury et al., 2024). Together, these data support the interpretation that mitochondrial fusion deficiency is associated with alterations in metabolic state, without establishing a direct causal link between mitochondrial morphology and metabolic function.

### Role of mitofusins in maintaining mitochondrial protein homeostasis

Beyond metabolic pathway remodeling, mitochondrial fusion may contribute to maintaining protein composition and stoichiometric balance within the mitochondrial network. For example, we observed decreased levels of SDHB and CACT, whereas other components of Complex II (SDHA) or β-oxidation (CPT2) were unaffected. Rather than indicating selective transcriptional regulation, these imbalances may reflect impaired assembly, stability, or distribution of proteins in the absence of fusion. Fusion enables exchange and homogenization of mitochondrial contents, buffering against local deficiencies and supporting protein quality control (Haroon and Vermulst, 2016). In the absence of fusion, fragmented mitochondria may exhibit impaired distribution or stability of specific proteins, potentially contributing to the observed imbalances. Similar disruptions in mitochondrial dynamics and protein homeostasis have been implicated in neurodegenerative diseases, including Charcot–Marie–Tooth disease (Züchner et al., 2004), Parkinson’s disease (Poole et al., 2008), Huntington’s disease (Shirendeb et al., 2011), and Alzheimer’s disease (Wang et al., 2009), suggesting that this mechanism may represent a broader vulnerability of neurons.

### Induced cellular stress by mitochondrial abnormality and compensatory biological reaction

Gene set enrichment analysis revealed significant upregulation of pathways related to ER stress and UPR in *Rho-cre/Mfn1* ^*flx/flx*^ */Mfn2* ^*flx/flx*^ mice (Figures 3A,B). ER stress can be triggered by multiple cellular stressors, including oxidative and metabolic stress (Ron and Walter, 2007; Rath and Haller, 2011; Chong et al., 2017). Our results suggest that mitochondrial fusion defects are associated with activation of ER stress pathways, which was also observed in another model (Ngoh et al., 2012). Since ER stress accumulates damage to cells and causes apoptosis (Iurlaro and Muñoz-Pinedo, 2016), various cytoprotective pathways exist to mitigate it. Biosynthesis of specific amino acids and cognate tRNA synthetases have been reported as biological processes to relieve ER stress (Gonen et al., 2019). Our gene expression analyses suggest that *Rho-cre/Mfn1* ^*flx/flx*^ */Mfn2* ^*flx/flx*^ mice exhibit an upregulation of amino acid metabolism and translation pathways including tRNA synthesis in response to cellular stress. This coordinated increase in protein translation is reminiscent of patterns observed in neurodegenerative diseases such as Huntington’s disease (Creus-Muncunill et al., 2019). Under ER stress and oxidative stress, translational reprogramming is mediated by stress response factors, including activating transcription factor 3 (ATF3), ATF4, and C/EBP homologous protein (CHOP) to preserve cellular homeostasis (Harding et al., 2003; Oyadomari and Mori, 2004; Costa-Mattioli and Walter, 2020; Ku and Cheng, 2020). Our RNA-seq data showed significantly elevated expression of these transcription factors (Figure 3C), suggesting that translational reprograming is activated as an adaptive mechanism to counteract the cellular stress.

The mTORC1 pathway, which shows increased activity in *Rho-cre/Mfn1* ^*flx/flx*^ */Mfn2* ^*flx/flx*^ mice (Figures 3C, 5G), is known to regulate a range of biosynthetic processes (Takei and Nawa, 2014; Valvezan et al., 2017). mTORC1 has been implicated in the regulation of mitochondrial biogenesis, nucleotide synthesis, and translation under conditions of cellular stress (Panwar et al., 2023). Consistent with this, our metabolomics and RNA-seq analyses revealed increased levels of metabolites and transcripts related to nucleotide synthesis and translation in *Rho-cre/Mfn1* ^*flx/flx*^ */Mfn2* ^*flx/flx*^ mice (Figures 3B, 4B,C). mTORC1 activity is influenced by amino acid metabolism and oxidative stress, both of which are linked to mitochondrial stress (Saxton and Sabatini, 2017; Li and Hoppe, 2023; Panwar et al., 2023), in line with our observations. Metabolomics analysis also identified changes in metabolites associated with mTORC1 activity, including DHAP (Figures 4B,D) (Gu et al., 2020; Otto, 2020). In addition, mitochondrial dysfunction is commonly associated with increased production of reactive oxygen species (ROS), which can contribute to cellular stress. The elevated glutathione disulfide (GSSG) levels observed in *Rho-cre/Mfn1* ^*flx/flx*^ */Mfn2* ^*flx/flx*^ mice (Figure 4B) are consistent with altered redox balance. Glutathione (GSH), a key cellular antioxidant composed of amino acids, is oxidized to GSSG during redox regulation (Georgiou-Siafis and Tsiftsoglou, 2023; Jomova et al., 2024), consistent with increased oxidative stress in these cells. Mitochondrial oxidative stress has also been linked to ER stress (Cao and Kaufman, 2014). Together, these findings are consistent with cellular responses associated with mitochondrial stress. Despite these adaptive or compensatory changes, chronic mitochondrial fusion deficiency is associated with progressive photoreceptor degeneration.

### Limitation of our study

In our study, we used protein abundance analysis to identify changes in the expression of mitochondrial metabolism related proteins in response to mitochondrial morphological dysfunction. We found that failure of mitochondrial fusion is associated with decreased expression of several proteins involved in OXPHOS, glycolysis, and mitochondrial β-oxidation. However, the lysates used in these studies contain proteins from all cells in the neural retina. Therefore, our analysis may underestimate the changes in protein expression that occur within rod photoreceptor cells, and more genes and proteins may be altered in these cells. ATP measurements were performed using whole neural retina lysates. Because MFN1 and MFN2 were selectively ablated in rod photoreceptors, changes in total retinal ATP levels may underestimate rod-specific bioenergetic alterations. Alternatively, these measurements may reflect contributions from multiple retinal cell types, and thus we cannot definitively attribute the observed changes solely to rod photoreceptors. In future studies, analyses employing a single-cell approach specific to rod photoreceptor cells would be very beneficial as it would allow a deeper exploration of other key players that respond to defects in mitochondrial fusion. In addition, we did not directly assess mitochondrial fusion and fission dynamics or the activity of fission-related proteins such as DRP1. Therefore, we cannot distinguish whether the observed mitochondrial fragmentation reflects reduced fusion, increased fission, or a combination of both. Future studies examining the balance of fusion and fission processes will be important to further define the underlying mechanisms.

## Conclusion

In conclusion, our study demonstrates that mitochondrial fusion mediated by MFN1 and MFN2 is essential for establishing and maintaining the specialized mitochondrial architecture of rod photoreceptor cells. Loss of mitochondrial fusion is associated with alterations in molecular pathways, early functional impairment, and progressive degeneration. While mitochondrial structural disruption, molecular remodeling, and functional decline occur in parallel, these findings reflect multiple aspects of the phenotypes associated with impaired mitochondrial fusion rather than a direct causal sequence. Together, these results provide insight into how mitochondrial fusion contributes to photoreceptor homeostasis and, given the increasingly recognized contribution of mitochondrial dysfunction in aging and neurodegenerative diseases (Eells, 2019; Ferrington et al., 2020), may further inform our understanding of mitochondrial dysfunction in retinal and neurodegenerative diseases.

## Data Availability

The datasets presented in this study can be found in online repositories. The names of the repository/repositories and accession number(s) can be found below: https://www.ncbi.nlm.nih.gov/, GSE297370 https://www.ebi.ac.uk/metabolights/, MTBLS12512.

## Glossary

3D-PCA: 3-dimensional principal component analysis
AA: amino acids
ADP: adenosine diphosphate
AMP: adenosine monophosphate
ATF3: activating transcription factor 3
ATF4: activating transcription factor 4
ATP: adenosine triphosphate
ATP5A: ATP synthase F1 subunit alpha
AvgExpr: average expression
CI: Complex I
CII: Complex II
CIII: Complex III
CIV: Complex IV
CV: Complex V
C/EBPγ: CCAAT/enhancer-binding protein Gamma
CACT: carnitine-acylcarnitine translocase
Cho: choroid
cKO: conditional knockout
CoA: coenzyme A
Cone: cone photoreceptors
CHOP: C/EBP homologous protein
CPT2: carnitine palmitoyl transferase II
DHAP: dihydroxyacetone phosphate
EM: electron microscopy
ER: endoplasmic reticulum
FAD: Flavin adenine dinucleotide
FASN: fatty acid synthase
FC: fold change
FDR: false discovery rate
Fis1: Fission Mitochondrial 1
GAP: glyceraldehyde-3-phosphate
GAPDH: glyceraldehyde-3-phosphate dehydrogenase
GDP: guanosine diphosphate
GMP: guanosine monophosphate
GSEA: gene set enrichment analysis
GSH: glutathione
GSSG: glutathione disulfide
GTP: guanosine triphosphate
H&E: hematoxylin and eosin
HR2: C-terminal heptad repeat domain
IMM: inner mitochondrial membrane
IMP: inosine monophosphate
INL: inner nuclear layer
IPL: inner plexiform layer
IS: inner segments
LDH: lactate dehydrogenase
Mac: macrophages
Mfn1: Mitofusin 1
Mfn2: Mitofusin 2
Mfns: Mitofusins
MTCO1: mitochondrially encoded cytochrome c oxidase I
mTOR: mammalian target of rapamycin
NDUFB8: NADH dehydrogenase [ubiquinone] 1 beta subcomplex subunit 8
NES: normalized enrichment score
OligoDend: oligodendrocytes
OMM: outer mitochondrial membrane
ONL: outer nuclear layer
ONLT: outer nuclear layer thickness
OPA: OPA1 mitochondrial dynamin like GTPase
OPL: outer plexiform layer
OS: outer segments
OXPHOS: oxidative phosphorylation
PFA: paraformaldehyde
PKM2: pyruvate kinase M2
PMP70: 70-kDa peroxisomal membrane protein
p-mTOR: phosphorylated mammalian target of rapamycin
PRPP: phosphoribosyl pyrophosphate
RGC: retinal ganglion cells
Rho: rhodopsin
RNA-seq: RNA-sequencing
Rod: rod photoreceptors
ROS: reactive oxygen species
RPE: retinal pigment epithelium
SDHA: succinate dehydrogenase A
SDHB: succinate dehydrogenase B
TCA: tricarboxylic acid
TOMM20: translocase of outer mitochondrial membrane 20
tRNA: transfer RNA
TUB: alpha-tubulin
UDP: uridine diphosphate
UMP: uridine monophosphate
UPR: unfolded protein response
UTP: uridine triphosphate
UQCRC2: ubiquinol-cytochrome c reductase core protein 2
XMP: xanthosine monophosphate

## References

[B1] Adebayo M. Singh S. Singh A. P. Dasgupta S. (2021). Mitochondrial fusion and fission: the fine-tune balance for cellular homeostasis. FASEB J. 35, e21620. 10.1096/fj.202100067R 34048084 PMC8415099

[B3] Ames 3rd A. Li Y.-Y. Heher E. C. Kimble C. R. (1992). Energy metabolism of rabbit retina as related to function: high cost of Na+ transport. J. Neurosci. 12, 840–853. 10.1523/JNEUROSCI.12-03-00840.1992 1312136 PMC6576058

[B4] Bauer M. F. Hofmann S. Neupert W. Brunner M. (2000). Protein translocation into mitochondria: the role of TIM complexes. Trends Cell Biol. 10, 25–31. 10.1016/s0962-8924(99)01684-0 10603473

[B5] Cao S. S. Kaufman R. J. (2014). Endoplasmic reticulum stress and oxidative stress in cell fate decision and human disease. Antioxid. Redox Signal. 21, 396–413. 10.1089/ars.2014.5851 24702237 PMC4076992

[B6] Chen H. McCaffery J. M. Chan D. C. (2007). Mitochondrial fusion protects against neurodegeneration in the cerebellum. Cell 130, 548–562. 10.1016/j.cell.2007.06.026 17693261

[B7] Chen W. Zhao H. Li Y. (2023a). Mitochondrial dynamics in health and disease: mechanisms and potential targets. Signal Transduct. Target. Ther. 8, 333. 10.1038/s41392-023-01547-9 37669960 PMC10480456

[B8] Chen Y. Zizmare L. Calbiague V. Wang L. Yu S. Herberg F. W. (2023b). Retinal metabolism displays evidence for uncoupling of glycolysis and oxidative phosphorylation *via* cori-cahill-and mini-krebs-cycle. Elife 12, RP91141. 10.7554/eLife.91141 38739438 PMC11090511

[B9] Chong W. C. Shastri M. D. Eri R. (2017). Endoplasmic Reticulum stress and oxidative stress: a vicious Nexus implicated in bowel disease pathophysiology. Int. J. Mol. Sci. 18, 771. 10.3390/ijms18040771 28379196 PMC5412355

[B10] Chong J. Soufan O. Li C. Caraus I. Li S. Bourque G. (2018). MetaboAnalyst 4.0: towards more transparent and integrative metabolomics analysis. Nucleic Acids Res. 46, W486–W494. 10.1093/nar/gky310 29762782 PMC6030889

[B11] Choudhury D. Rong N. Senthil Kumar H. V. Swedick S. Samuel R. Z. Mehrotra P. (2024). Proline restores mitochondrial function and reverses aging hallmarks in senescent cells. Cell Rep. 43, 113738. 10.1016/j.celrep.2024.113738 38354087 PMC13092368

[B12] Costa-Mattioli M. Walter P. (2020). The integrated stress response: from mechanism to disease. Science 368, eaat5314. 10.1126/science.aat5314 32327570 PMC8997189

[B13] Creus-Muncunill J. Badillos-Rodrı´guez R. Garcia-Forn M. Masana M. ` Barriga G.G.-D. ´az Guisado-Corcoll A. (2019). Increased translation as a novel pathogenic mechanism in Huntington’s disease. Brain 142, 3158–3175. 10.1093/brain/awz274 31365052

[B14] Dai W. Jiang L. (2019). Dysregulated mitochondrial dynamics and metabolism in obesity, diabetes, and cancer. Front. Endocrinol. (Lausanne) 10, 570. 10.3389/fendo.2019.00570 31551926 PMC6734166

[B15] Dash S. Dash C. Pandhare J. (2021). Activation of proline metabolism maintains ATP levels during cocaine-induced polyADP-ribosylation. Amino Acids 53, 1903–1915. 10.1007/s00726-021-03065-w 34417893 PMC8651605

[B16] Eells J. T. (2019). Mitochondrial dysfunction in the aging retina. Biol. (Basel) 8, 31. 10.3390/biology8020031 31083549 PMC6627398

[B17] Ferrington D. A. Fisher C. R. Kowluru R. A. (2020). Mitochondrial defects drive degenerative retinal diseases. Trends Mol. Med. 26, 105–118. 10.1016/j.molmed.2019.10.008 31771932 PMC6938541

[B18] Filadi R. Pendin Di. Pizzo P. (2018). Mitofusin 2: from functions to disease. Cell Death Dis. 9, 330. 10.1038/s41419-017-0023-6 29491355 PMC5832425

[B19] Fu Z. Kern T. S. Hellström A. Smith L. E. H. (2021). Fatty acid oxidation and photoreceptor metabolic needs. J. Lipid Res. 62, 100035. 10.1194/jlp.TR120000618 32094231 PMC7905050

[B20] Georgiou-Siafis S. K. Tsiftsoglou A. S. (2023). The key role of GSH in keeping the redox balance in Mammalian cells: mechanisms and significance of GSH in detoxification *via* Formation of conjugates. Antioxidants (Basel) 12, 1953. 10.3390/antiox12111953 38001806 PMC10669396

[B21] Gonen N. Meller A. Sabath N. Shalgi R. (2019). Amino acid biosynthesis regulation during endoplasmic reticulum stress is coupled to protein expression demands. iScience 19, 204–213. 10.1016/j.isci.2019.07.022 31377665 PMC6698312

[B22] Gu Z. Liu Y. Qiu B. Liu H. Fang W. (2020). Risk stratification is helpful in designing Follow-Up strategy and future studies on adjuvant therapies: response to the external validation on the Chinese alliance for research in Thymomas predictive model of recurrence. J. Thorac. Oncol. 15, e139–e141. 10.1016/j.jtho.2020.06.010 32718540

[B23] Guimarães-Ferreira L. (2014). Role of the phosphocreatine system on energetic homeostasis in skeletal and cardiac muscles. Einstein (Sao Paulo) 12, 126–131. 10.1590/s1679-45082014rb2741 24728259 PMC4898252

[B24] Harding H. P. Zhang Y. Zeng H. Novoa I. Lu P. D. Calfon M. (2003). An integrated stress response regulates amino acid metabolism and resistance to oxidative stress. Mol. Cell 11, 619–633. 10.1016/s1097-2765(03)00105-9 12667446

[B25] Haroon S. Vermulst M. (2016). Linking mitochondrial dynamics to mitochondrial protein quality control. Curr. Opin. Genet. Dev. 38, 68–74. 10.1016/j.gde.2016.04.004 27235806

[B26] Hetz C. (2012). The unfolded protein response: controlling cell fate decisions under ER stress and beyond. Nat. Rev. Mol. Cell Biol. 13, 89–102. 10.1038/nrm3270 22251901

[B27] Huggins C. J. Mayekar M. K. Martin N. Saylor K. L. Gonit M. Jailwala P. (2016). C/EBPγ is a critical regulator of cellular stress response networks through heterodimerization with ATF4. Mol. Cell Biol. 36, 693–713. 10.1128/MCB.00911-15 26667036 PMC4760225

[B28] Ibrahim D. R. Schwarz K. Suiwal S. Maragkou S. Schmitz F. (2025). Early synapse-specific alterations of Photoreceptor Mitochondria in the EAE mouse model of multiple sclerosis. Cells 14, 206. 10.3390/cells14030206 39936997 PMC11816939

[B29] Ishihara N. Eura Y. Mihara K. (2004). Mitofusin 1 and 2 play distinct roles in mitochondrial fusion reactions *via* GTPase activity. J. Cell Sci. 117, 6535–6546. 10.1242/jcs.01565 15572413

[B30] Iurlaro R. Muñoz-Pinedo C. (2016). Cell death induced by endoplasmic reticulum stress. FEBS J. 283, 2640–2652. 10.1111/febs.13598 26587781

[B31] Jiao X. Ma Z. Lei J. Liu P. Cai X. Shahi P. K. (2022). Retinal development and pathophysiology in Kcnj13 knockout mice. Front. Cell Developmental Biology 9, 810020. 10.3389/fcell.2021.810020 35096838 PMC8790323

[B32] Johnson B. A. Ikeda S. Pinto L. H. Ikeda A. (2006). Reduced synaptic vesicle density and aberrant synaptic localization caused by a splice site mutation in the Rs1h gene. Vis. Neurosci. 23, 887–898. 10.1017/S0952523806230244 17266781

[B33] Jomova K. Alomar S. Y. Alwasel S. H. Nepovimova E. Kuca K. Valko M. (2024). Several lines of antioxidant defense against oxidative stress: antioxidant enzymes, nanomaterials with multiple enzyme-mimicking activities, and low-molecular-weight antioxidants. Arch. Toxicol. 98, 1323–1367. 10.1007/s00204-024-03696-4 38483584 PMC11303474

[B34] Kemp F. Braverman E. L. Byersdorfer C. A. (2024). Fatty acid oxidation in immune function. Front. Immunol. 15, 1420336. 10.3389/fimmu.2024.1420336 39007133 PMC11240245

[B35] Ku H. C. Cheng C. F. (2020). Master Regulator activating transcription factor 3 (ATF3) in metabolic homeostasis and cancer. Front. Endocrinol. (Lausanne) 11, 556. 10.3389/fendo.2020.00556 32922364 PMC7457002

[B36] Lee W.-H. Higuchi H. Ikeda S. Macke E. L. Takimoto T. Pattnaik B. R. (2016). Mouse Tmem135 mutation reveals a mechanism involving mitochondrial dynamics that leads to age-dependent retinal pathologies. Elife 5, e19264. 10.7554/eLife.19264 27863209 PMC5117855

[B37] Lewis S. A. Takimoto T. Mehrvar S. Higuchi H. Doebley A. L. Stokes G. (2018). The effect of Tmem135 overexpression on the mouse heart. PLoS One 13, e0201986. 10.1371/journal.pone.0201986 30102730 PMC6089435

[B38] Li Q. Hoppe T. (2023). Role of amino acid metabolism in mitochondrial homeostasis. Front. Cell Dev. Biol. 11, 1127618. 10.3389/fcell.2023.1127618 36923249 PMC10008872

[B39] Li S. Sheng Z. H. (2022). Energy matters: presynaptic metabolism and the maintenance of synaptic transmission. Nat. Rev. Neurosci. 23, 4–22. 10.1038/s41583-021-00535-8 34782781

[B40] Liao Y. Wang J. Jaehnig E. J. Shi Z. Zhang B. (2019). WebGestalt 2019: gene set analysis toolkit with revamped UIs and APIs. Nucleic Acids Res. 47, W199–W205. 10.1093/nar/gkz401 31114916 PMC6602449

[B41] Licata L. Lo Surdo P. Iannuccelli M. Palma A. Micarelli E. Perfetto L. (2020). SIGNOR 2.0, the SIGnaling Network Open Resource 2.0: 2019 update. Nucleic Acids Res. 48, D504–D510. 10.1093/nar/gkz949 31665520 PMC7145695

[B42] Linton J. D. Holzhausen L. C. Babai N. Song H. Miyagishima K. J. Stearns G. W. (2010). Flow of energy in the outer retina in darkness and in light. Proc. Natl. Acad. Sci. U. S. A. 107, 8599–8604. 10.1073/pnas.1002471107/-/DCSupplemental 20445106 PMC2889335

[B43] Meschede I. P. Ovenden N. C. Seabra M. C. Futter C. E. Votruba M. Cheetham M. E. (2020). Symmetric arrangement of mitochondria:plasma membrane contacts between adjacent photoreceptor cells regulated by Opa1. Proc. Natl. Acad. Sci. U. S. A. 117, 15684–15693. 10.1073/pnas.2000304117 32571921 PMC7355040

[B44] Misko A. Jiang S. Wegorzewska I. Milbrandt J. Baloh R. H. (2010). Mitofusin 2 is necessary for transport of axonal mitochondria and interacts with the Miro/Milton complex. J. Neurosci. 30, 4232–4240. 10.1523/JNEUROSCI.6248-09.2010 20335458 PMC2852190

[B45] Muñ oz-Pinedo C. Guío-Carrió A. Goldstein J. C. Fitzgerald P. Newmeyer D. D. Green D. R. (2006). Different mitochondrial intermembrane space proteins are released during apoptosis in a manner that is coordinately initiated but can vary in duration. Proc. Natl. Acad. Sci. U. S. A. 103, 11573–11578. 10.1073/pnas.0603007103 16864784 PMC1518810

[B46] Narayan D. S. Chidlow G. Wood J. P. M. Casson R. J. (2017). Glucose metabolism in mammalian photoreceptor inner and outer segments. Clin. Exp. Ophthalmol. 45, 730–741. 10.1111/ceo.12952 28334493

[B47] Ngoh G. A. Papanicolaou K. N. Walsh K. (2012). Loss of mitofusin 2 promotes endoplasmic reticulum stress. J. Biol. Chem. 287, 20321–20332. 10.1074/jbc.M112.359174 22511781 PMC3370214

[B48] Oikawa K. Torne O. Sun D. Moon A. K. B. Kiland J. A. Trane R. M. (2023). Aqueous humor TGF-β2 and its Association with intraocular pressure in a naturally occurring large animal model of glaucoma. Investigative Ophthalmology and Visual Science 64 (10), 18. 10.1167/iovs.64.10.18 37459065 PMC10362923

[B49] Otto A. M. (2020). Small is beautiful–a glycolytic metabolite signals mTORC1 activation in cancer cell metabolism. Signal Transduct. Target. Ther. 5, 259. 10.1038/s41392-020-00371-9 33144567 PMC7609526

[B50] Oyadomari S. Mori M. (2004). Roles of CHOP/GADD153 in endoplasmic reticulum stress. Cell Death Differ. 11, 381–389. 10.1038/sj.cdd.4401373 14685163

[B51] Ozaki T. Utsumi S. Iwamoto T. Tanaka M. Tomita H. Sugano E. (2020). Data on mitochondrial ultrastructure of photoreceptors in pig, rabbit, and mouse retinas. Data Brief. 30, 105544. 10.1016/j.dib.2020.105544 32368587 PMC7186507

[B52] Pan W. W. Wubben T. J. Besirli C. G. (2021). Photoreceptor metabolic reprogramming: current understanding and therapeutic implications. Commun. Biol. 4, 245. 10.1038/s42003-021-01765-3 33627778 PMC7904922

[B53] Panwar V. Singh A. Bhatt M. Tonk R. K. Azizov S. Raza A. S. (2023). Multifaceted role of mTOR (mammalian target of rapamycin) signaling pathway in human health and disease. Signal Transduct. Target. Ther. 8, 375. 10.1038/s41392-023-01608-z 37779156 PMC10543444

[B54] Poole A. C. Thomas R. E. Andrews L. A. Mcbride H. M. Whitworth A. J. Pallanck L. J. (2008). The PINK1/Parkin pathway regulates mitochondrial morphology. Proc. Natl. Acad. Sci. U. S. A. 105, 1638–1643. 10.1073/pnas.0709336105 18230723 PMC2234197

[B55] Ramírez S. Gómez-Valadés A. G. Schneeberger M. Varela L. Haddad-Tóvolli R. Altirriba J. (2017). Mitochondrial dynamics mediated by mitofusin 1 is required for POMC neuron glucose-sensing and insulin release control. Cell Metab. 25, 1390–1399.e6. 10.1016/j.cmet.2017.05.010 28591639

[B56] Rath E. Haller D. (2011). Inflammation and cellular stress: a mechanistic link between immune-mediated and metabolically driven pathologies. Eur. J. Nutr. 50, 219–233. 10.1007/s00394-011-0197-0 21547407

[B57] Robinson M. D. McCarthy D. J. Smyth G. K. (2009). edgeR: a Bioconductor package for differential expression analysis of digital gene expression data. Bioinformatics 26, 139–140. 10.1093/bioinformatics/btp616 19910308 PMC2796818

[B58] Ron D. Walter P. (2007). Signal integration in the endoplasmic reticulum unfolded protein response. Nat. Rev. Mol. Cell Biol. 8, 519–529. 10.1038/nrm2199 17565364

[B59] Santel A. Fuller M. T. (2001). Control of mitochondrial morphology by a human mitofusin. J. Cell. Sci. 114, 867–874. 10.1242/jcs.114.5.867 11181170

[B60] Saxton R. A. Sabatini D. M. (2017). mTOR signaling in growth, metabolism, and disease. Cell 169, 361–371. 10.1016/j.cell.2017.03.035 28388417

[B61] Schneeberger M. Dietrich M. O. Sebastián D. Imbernón M. Castaño C. Garcia A. (2013). Mitofusin 2 in POMC neurons connects ER stress with leptin resistance and energy imbalance. Cell 155, 172–187. 10.1016/j.cell.2013.09.003 24074867 PMC3839088

[B62] Schrepfer E. Scorrano L. (2016). Mitofusins, from Mitochondria to metabolism. Mol. Cell 61, 683–694. 10.1016/j.molcel.2016.02.022 26942673

[B63] Shahin S. Lu B. Zhou Y. Xu H. Chetsawang J. Baloh R. H. (2023). MFN1 augmentation prevents retinal degeneration in a charcot-marie-tooth type 2A mouse model. iScience 26, 106270. 10.1016/j.isci.2023.106270 36936780 PMC10014277

[B64] Shirendeb U. Reddy A. P. Manczak M. Calkins M. J. Mao P. Tagle D. A. (2011). Abnormal mitochondrial dynamics, mitochondrial loss and mutant huntingtin oligomers in Huntington’s disease: implications for selective neuronal damage. Hum. Mol. Genet. 20, 1438–1455. 10.1093/hmg/ddr024 21257639 PMC3049363

[B65] Tábara L. C. Segawa M. Prudent J. (2025). Molecular mechanisms of mitochondrial dynamics. Nat. Rev. Mol. Cell Biol. 26, 123–146. 10.1038/s41580-024-00785-1 39420231

[B66] Takei N. Nawa H. (2014). mTOR signaling and its roles in normal and abnormal brain development. Front. Mol. Neurosci. 7, 28. 10.3389/fnmol.2014.00028 24795562 PMC4005960

[B67] Tilokani L. Nagashima S. Paupe V. Prudent J. (2018). Mitochondrial dynamics: overview of molecular mechanisms. Essays Biochem. 62, 341–360. 10.1042/EBC20170104 30030364 PMC6056715

[B68] Valvezan A. J. Turner M. Belaid A. Lam H. C. Miller S. K. McNamara M. C. (2017). mTORC1 couples nucleotide synthesis to nucleotide demand resulting in a targetable metabolic vulnerability. Cancer Cell 32, 624–638.e5. 10.1016/j.ccell.2017.09.013 29056426 PMC5687294

[B69] Vevea J. D. Chapman E. R. (2023). Mitofusin 2 sustains the axonal mitochondrial network to support presynaptic Ca21 homeostasis and the synaptic vesicle cycle in Rat hippocampal axons. J. Neurosci. 43, 3421–3438. 10.1523/JNEUROSCI.1356-22.2023 36997314 PMC10175236

[B70] Wang X. Su B. Lee H. G. Li X. Perry G. Smith M. A. (2009). Impaired balance of mitochondrial fission and fusion in Alzheimer’s disease. J. Neurosci. 29, 9090–9103. 10.1523/JNEUROSCI.1357-09.2009 19605646 PMC2735241

[B71] Xu F. Wang L. (2024). Deciphering ER stress-unfolded protein response relationship by visualizing unfolded proteins in the ER. Cell Rep. 43, 114358. 10.1016/j.celrep.2024.114358 38865243

[B72] Youle R. J. Van Der Bliek A. M. (2012). Mitochondrial Fission, fusion, and stress. Science 337, 1062–1065. 10.1126/science.1219855 22936770 PMC4762028

[B73] Zhou G. Soufan O. Ewald J. Hancock R. E. W. Basu N. Xia J. (2019). NetworkAnalyst 3.0: a visual analytics platform for comprehensive gene expression profiling and meta-analysis. Nucleic Acids Res. 47, W234–W241. 10.1093/nar/gkz240 30931480 PMC6602507

[B74] Züchner S. Mersiyanova I. V. Muglia M. Bissar-Tadmouri N. Rochelle J. Dadali E. L. (2004). Mutations in the mitochondrial GTPase mitofusin 2 cause charcot-marie-tooth neuropathy type 2A. Nat. Genet. 36, 449–451. 10.1038/ng1341 15064763

