# Supplementary material for: Mitofusins are required for specialized mitochondrial morphology and function of rod photoreceptor cells
Source: Front Cell Dev Biol. 2026 Jun 3;14:1724328. doi: 10.3389/fcell.2026.1724328 (PMC13273400; doi:10.3389/fcell.2026.1724328)
Supplement: Supplementary file 1 [file DataSheet1.docx]

Supplementary Material

Mitofusins are required for specialized mitochondrial morphology and function of rod photoreceptor cells

Michael Landowski^1,2 #^, Ryo Hagimori^1,3 #^, Purnima Gogoi^1,2^, Pawan K. Shahi^2,4^, Kazuya Oikawa^2,5,6^, Vijesh J. Bhute^1,7^, Gillian J. McLellan^2,5,6^, Sakae Ikeda^1,2^, Ken-ichi Yamada^3^, Bikash R. Pattnaik^2,4,5^, Tetsuya Takimoto^8^, Akihiro Ikeda^1,2*^

^1^ Department of Medical Genetics, University of Wisconsin-Madison, Madison, Wisconsin, United States of America

^2^ McPherson Eye Research Institute, University of Wisconsin-Madison, Madison, Wisconsin, United States of America

^3^ Department of Molecular Pathobiology, Faculty of Pharmaceutical Sciences, Kyushu University, Fukuoka, Japan

^4^ Department of Pediatrics, University of Wisconsin-Madison, Madison, Wisconsin, United States of America

^5^ Department of Ophthalmology & Visual Sciences, University of Wisconsin-Madison, Madison, Wisconsin, United States of America

^6^ Department of Surgical Sciences, University of Wisconsin-Madison, Madison, Wisconsin, United States of America

^7^ Department of Chemical Engineering, Imperial College London, South Kensington, London, United Kingdom

^8^ Oncology Innovation Center, Fujita Health University, Toyoake, Aichi, Japan

**# Both authors contributed equally: Michael Landowski and Ryo Hagimori**

*** Correspondence:**Corresponding Author: Akihiro Ikeda
aikeda@wisc.edu


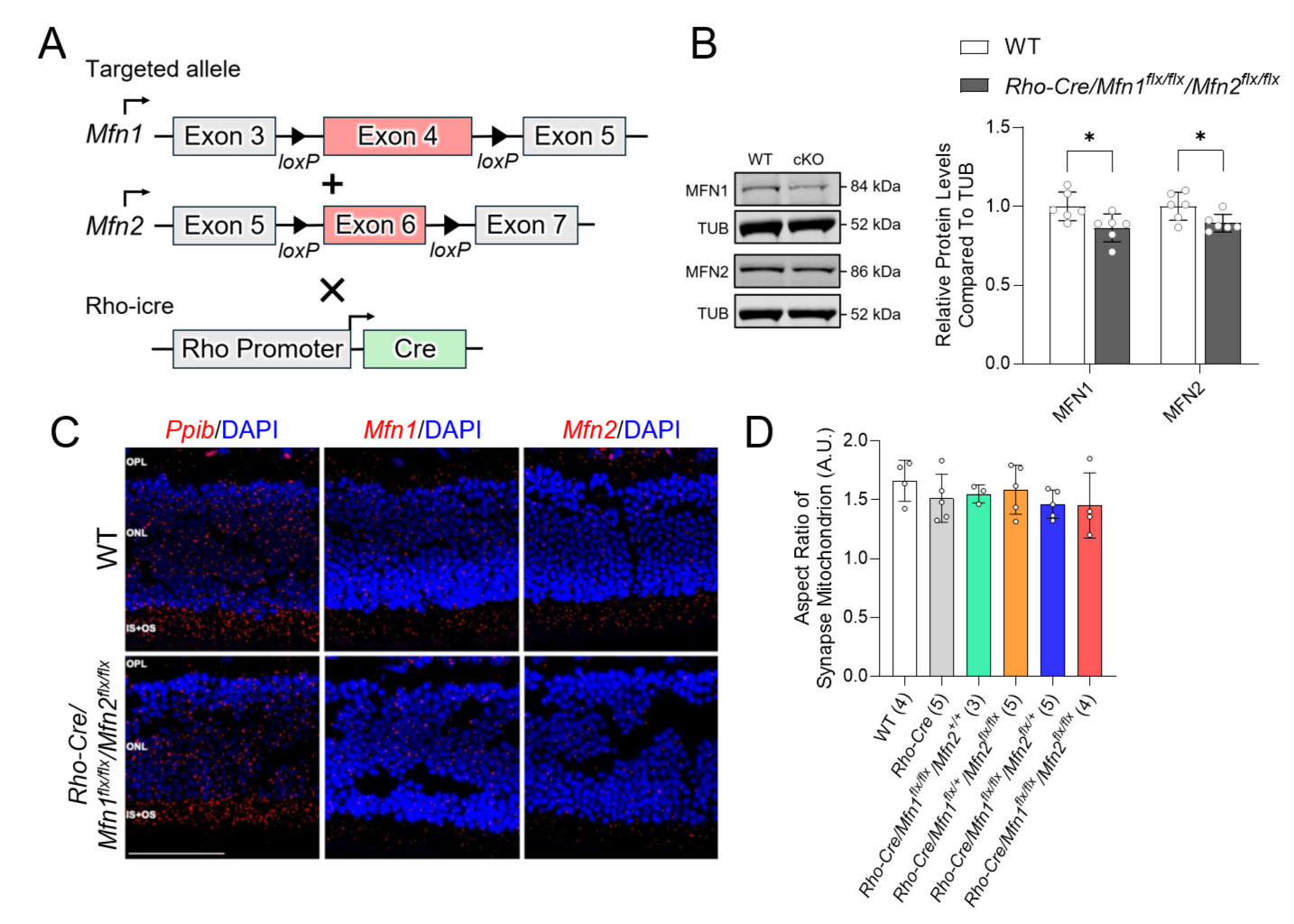


**Supplementary Figure 1.** **Combined ablation of *Mfn1* and *Mfn2* specifically in rod photoreceptor cells.**

(A) Schematic diagram of Cre-Lox strategy for rod photoreceptor cell-specific ablation of mitofusin1 (*Mfn1*) and mitofusin2 (*Mfn2*). (B) Western blot analysis of MFN1 and MFN2 using neural retinas from one-month-old WT and *Rho-Cre/Mfn1^flx/flx^/Mfn2^flx/flx^* mice. Alpha-tubulin (TUB) served as the loading control. Data are presented as mean ± SD. Asterisks (*) indicates P < 0.05 significance by t-test. Six one-month-old mice were used for each group. Dots represent individual data points. The protein size next to the immunoblot images denotes the size of the immunoband measured for this analysis. (C) Representative images of retinal cross-sections showing *Mfn1* and *Mfn2* transcripts detected by RNAscope ISH. Left panel: Abundant expression of *Ppib* (peptidylprolyl isomerase B) in WT (upper) and *Rho-Cre/Mfn1^flx/flx^/Mfn2^flx/flx^* (lower) retinas, as a positive control. Middle panel: Normal *Mfn1* expression in WT (upper) and a marked reduction of *Mfn1* signals in the *Rho-Cre/Mfn1^flx/flx^/Mfn2^flx/flx^* retina (lower). Right panel: Normal *Mfn2* expression in WT (upper) and a marked reduction of *Mfn2* signals in the *Rho-Cre/Mfn1^flx/flx^/Mfn2^flx/flx^* retina (lower). Magnification = 40X. Scale bar = 50 microns. (D) Quantification of the aspect ratio of mitochondria in the photoreceptor cell synaptic terminal. Number in the parenthesis denotes the number of mice used in the study. Data is presented as mean +/- SD and analyzed by one-way ANOVA with post-hoc Tukey’s test.

**Supplementary Figure 2.** Abnormal mitochondrial morphologies in rod photoreceptor synaptic terminals due to ablation of *Mfn1* and *Mfn2* at one month of age. Representative electron micrographs of rod photoreceptor synapses. Mitochondria are shaded in green. Magnification = 8,800X. Scale bar = 1 micron.

A

B

**
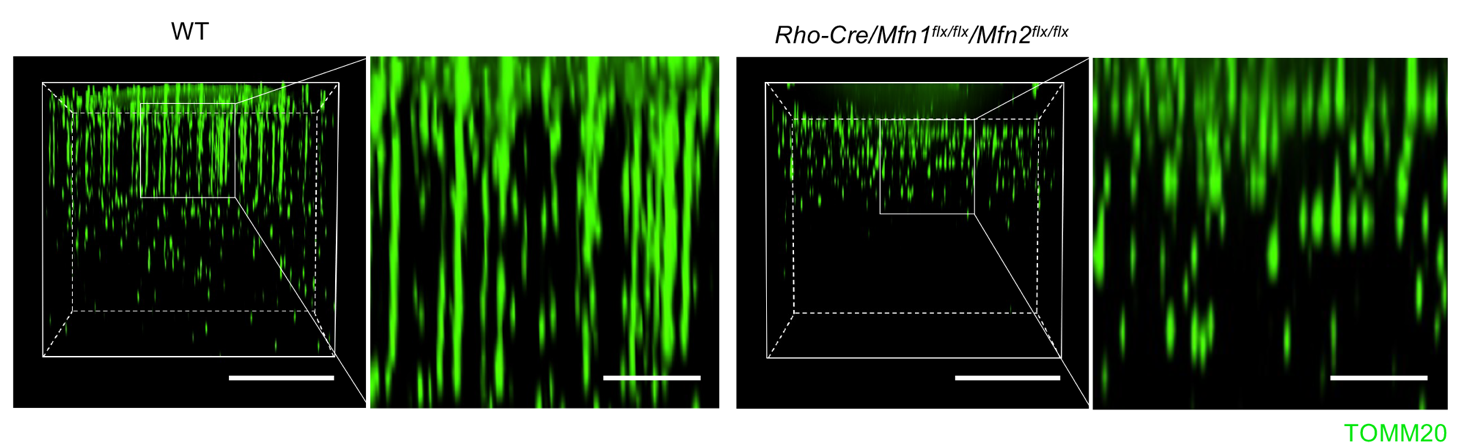
**

**Supplementary Figure 3.** Abnormal mitochondrial morphologies in rod photoreceptor inner segments due to ablation of *Mfn1* and *Mfn2* at one month of age. (A) Representative electron micrographs of rod photoreceptor inner segments. Mitochondria are shaded in blue. Magnification = 8,800X. Scale bar = 1 micron. (B) Representative three-dimensional reconstructions of mitochondria within photoreceptor inner segments, visualized by immunostaining with TOMM20. Scale bar = 20 μm; scale bar for magnified view = 5 μm.


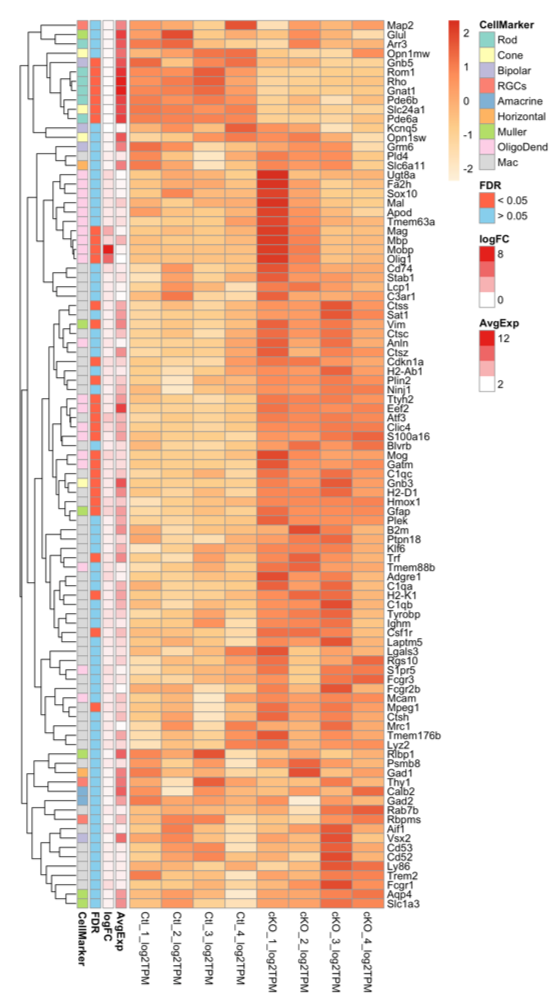


**Supplementary Figure 4. Gene expression analysis of specific markers for retinal cell types in mice with rod-specific ablation of *Mfn1* and *Mfn2***

Heatmap showing genes encoding specific markers for cells comprising the retina: rod photoreceptor cells (Rod), cone photoreceptor cells (Cone), bipolar cells, retinal ganglion cells (RGC), amacrine cells, horizontal cells, Müller cells, oligodendrocyte cells (OligoDend), macrophages (Mac). For each gene, false discovery rate (FDR), logFC, and average expression (AvgExpr) are shown in the left column.


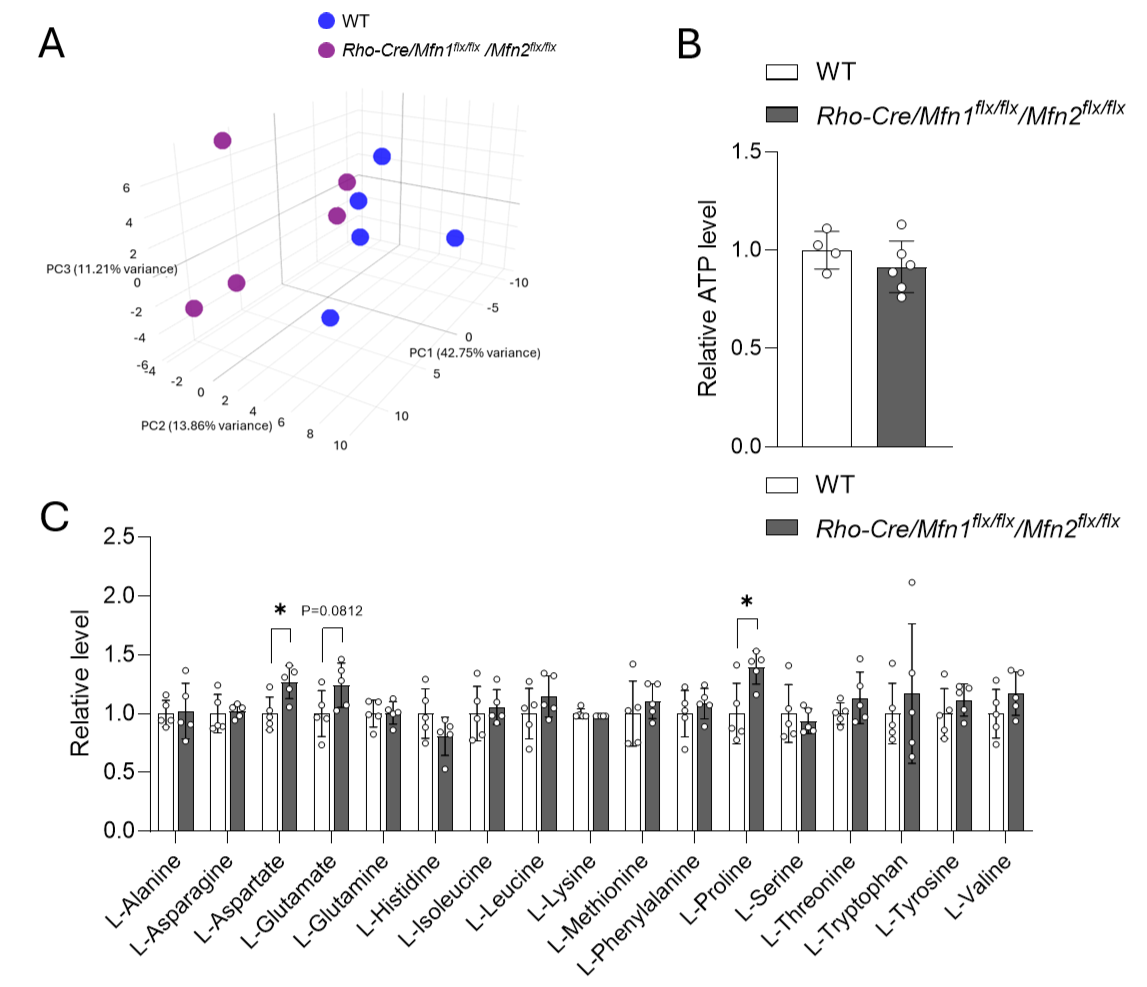


**Supplementary Figure 5. Three-dimensional principal component analysis and analysis of amino acid levels in the retina of mice with rod-specific ablation of *Mfn1* and *Mfn2***

(A) Three-dimensional principal component analysis of specialized metabolite components in *Rho-Cre/Mfn1^flx/flx^/Mfn2^flx/flx^* neural retinas compared to WT neural retinas. (B) ATP levels in neural retinas of WT and *Rho-Cre/Mfn1^flx/flx^/Mfn2^flx/flx^* mice. (C) Relative amino acid levels in the neural retina of *Rho-Cre/Mfn1^flx/flx^/Mfn2^flx/flx^* mice compared to WT mice. Data are presented as mean ± SD. Asterisks (*) indicate P < 0.05 significance by t-test. Five mice were used for each group in the study. Dots represent individual data points.


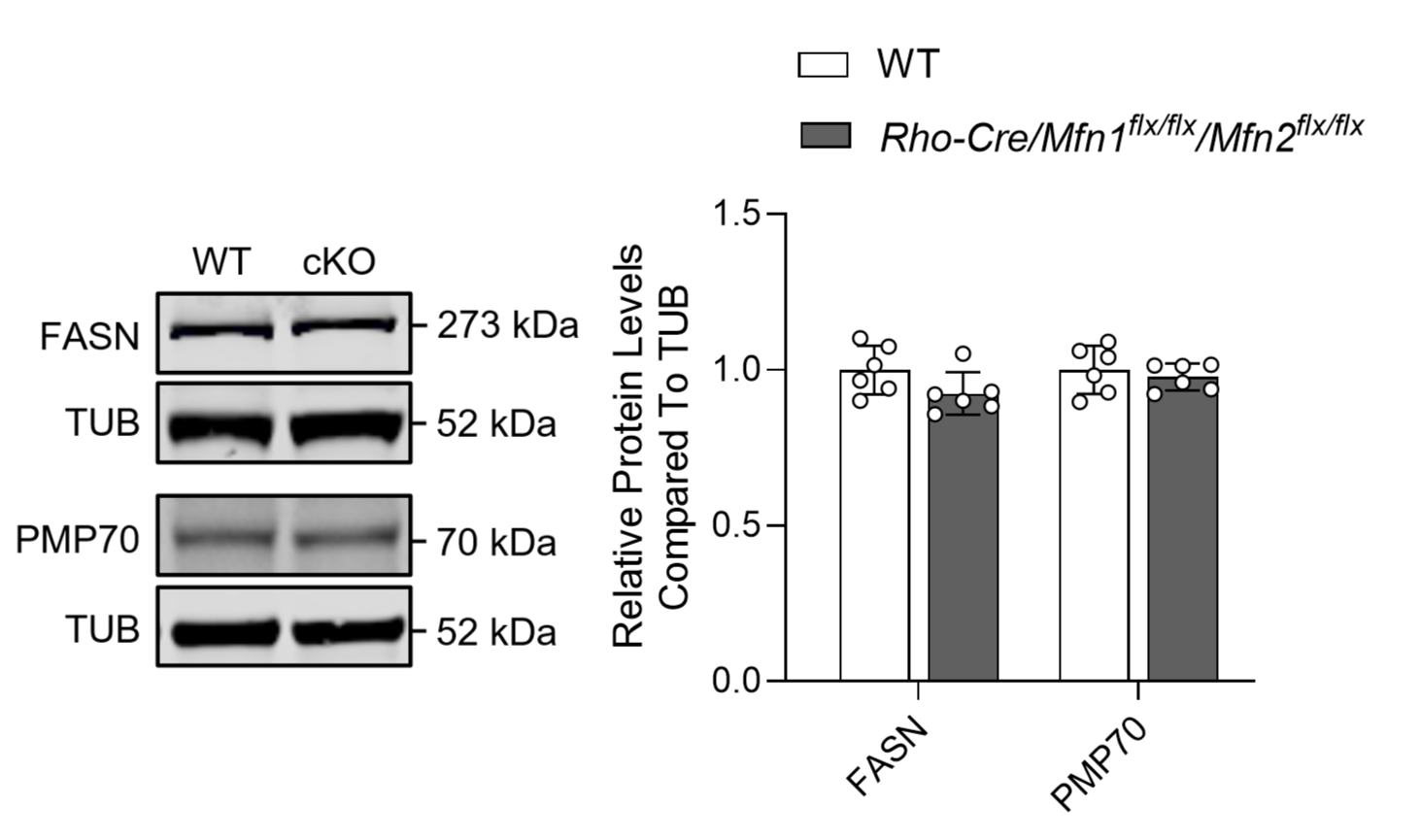


**Supplementary Figure 6. Levels of proteins related to peroxisomal β-oxidation in the retina of mice with rod-specific ablation of *Mfn1* and *Mfn2***

Western blot analysis of 70-kDa peroxisomal membrane protein (PMP70) and fatty acid synthase (FASN), related to peroxisomal β-oxidation in neural retinas from one-month-old WT and *Rho-Cre/Mfn1^flx/flx^/Mfn2^flx/flx^* mice. Alpha-tubulin (TUB) served as the loading control. Data are presented as mean ± SD. Asterisks (*) indicates P < 0.05 significance following a significant difference detected by t-test. Six one-month-old mice were used for each group in the study. Dots represent individual data points. The protein size next to the immunoblot images denotes the size of the immunoband measured for this analysis.
